# Cholestasis induced liver pathology results in dysfunctional immune responses after arenavirus infection

**DOI:** 10.1038/s41598-018-30627-y

**Published:** 2018-08-15

**Authors:** Elisabeth Lang, Vitaly I. Pozdeev, Prashant V. Shinde, Haifeng C. Xu, Balamurugan Sundaram, Yuan Zhuang, Gereon Poschmann, Jun Huang, Kai Stühler, Aleksandra A. Pandyra, Verena Keitel, Dieter Häussinger, Karl S. Lang, Philipp A. Lang

**Affiliations:** 1 Department of Gastroenterology, Hepatology, and Infectious Diseases, Heinrich-Heine-University Düsseldorf, Universitätsstrasse. 1, 40225 Düsseldorf, Germany; 2 Department of Molecular Medicine II, Medical Faculty, Heinrich Heine University, Universitätsstrasse. 1, 40225 Düsseldorf, Germany; 3 Molecular Proteomics Laboratory, Biomedical Research Center (BMFZ), Heinrich-Heine-Universität, Düsseldorf, Medical Faculty, Duesseldorf, Germany; 4 Institute of Immunology, Medical Faculty, University of Duisburg-Essen, Hufelandstrasse. 55, Essen, 45147 Germany; 5 Institute for Molecular Medicine, University Hospital Düsseldorf, Düsseldorf, Germany; 6 Laboratory of Oncolytic-Virus-Immuno-Therapeutics (LOVIT), German Cancer Research Center (DKFZ), Im Neunheimer Feld 242, 69120 Heidelberg, Germany; 7 Laboratory of Oncolytic-Virus-Immuno-Therapeutics (LOVIT), Luxembourg Institute of Health (LIH), 84, rue Val Fleuri, L-1526 Strassen, Luxembourg

## Abstract

Immune responses are critical for defense against pathogens. However, prolonged viral infection can result in defective T cell immunity, leading to chronic viral infection. We studied immune activation in response to arenavirus infection during cholestasis using bile duct ligation (BDL). We monitored T cell responses, virus load and liver pathology markers after infection with lymphocytic choriomeningitis virus (LCMV). BDL mice failed to induce protective anti-viral immunity against LCMV and consequently exhibited chronic viral infection. BDL mice exhibited reduced anti-viral T cell immunity as well as reduced type 1 interferon production early after LCMV infection. Consistently, the presence of serum from BDL mice reduced the responsiveness of dendritic cell (DC) and T cell cultures when compared to Sham controls. Following fractionation and mass spectrometry analyses of sera, we identified several serum factors to be upregulated following BDL including bilirubin, bile acids, 78 kDa Glucose regulated protein (GRP78) and liver enzymes. Bilirubin and GRP78 were capable of inhibiting DC and T cell activation. In this work, we demonstrate that liver damage mediated by cholestasis results in defective immune induction following arenavirus infection.

## Introduction

Failure to process bile acids from the liver by either intrahepatic or extrahepatic mechanisms can lead to acute cholestasis^1^. Factors leading to acute cholestasis include post-operative cholestasis following liver transplant, sepsis, drug intake, acute cholangitis, pregnancy and gallstones^2–4^. Viral infections such as Hepatitis A, B, C and E, as well as Epstein-Barr virus can trigger acute cholestasis in humans^5–8^. However, the effects of prior cholestasis on the course of an infection are not known. In mice, bile duct ligation (BDL) can be used to induce cholestasis, which causes constant liver damage and results in liver fibrosis within three weeks. This is accompanied by expression of collagens, alpha smooth muscle actin (a-SMA), and tissue inhibitor of metalloproteinase 1 (TIMP-1)^9–11^. During cholestasis mediated liver damage, bile acid accumulation induces expression of endoplasmic reticulum (ER) stress response proteins such as CCAAT-enhancer-binding protein homologous protein (CHOP) or glucose-regulated protein-78/immunoglobulin heavy chain-binding protein (GRP78/BiP)^12,13^. CHOP deficiency results in decreased liver damage and liver fibrosis following bile duct ligation^12^. Whole body CHOP deficient animals are viable^14^ while GRP78 deficient animals are embryonically lethal^15^. Liver specific GRP78 deficient mice exhibit steatohepatitis and exacerbate the liver damaging effects of CCl_4_, an effect that correlates with increased CHOP expression^16^.

The immune activation in turn, can affect anti-bacterial immunity by inducing type I interferon (IFN-I) expression^17^. IFN-I production relies on activation of pathogen recognition receptors, which induce IFN-I transcription via central transcription factors^18^. IFN-I bind to the IFN-I receptor (IFNAR), which initiates transcription of anti-viral defense genes via Janus kinase (JAK) and Signal Transducer and Activator of Transcription (STAT) signaling^19^. Consequently, IFNAR signaling is critical in limiting viral replication during an infection^20^. IFN-I activation of other immune cells and its involvement in autoimmunity has also been described^19^. IFN-I also influences virus specific T cell immunity and protects T cells from natural killer (NK) cell mediated attack^21,22^. However, IFN-I production can lead to exhaustion of anti-viral T cells causing the establishment of a chronic viral infection^23,24^. Mice infected with the lymphocytic choriomeningitis virus (LCMV) strains Clone 13 and Docile show upregulation of programmed death ligand-1 (PD-1) on cytotoxic CD8^+^ T cells and have diminished antiviral T cell immunity. This phenomenon is termed as “T cell exhaustion”, and results in chronic viral infection^25–27^. Several other factors including T-cell immunoglobulin and mucin-domain containing-3 (TIM-3), lymphocyte-activation gene 3 (Lag-3), and interleukin-10 (IL-10) have also been shown to be involved in establishing T cell exhaustion^28^. However, how liver damage affects liver-specific anti-viral immune activation against LCMV is poorly understood.

In this study we show that acute cholestasis during early stages following BDL results in defective anti-viral defense causing persistent LCMV infection. Specifically, anti-viral T cells are dysfunctional and show an exhausted state in BDL animals. Furthermore, innate IFN-I production is reduced following LCMV infection. Our data indicate that serum components can contribute to defective dendritic cell and T cell activation. We show that bilirubin and GRP78 can limit DC mediated cytokine production and T cell proliferation *in vitro*. This immune modulation was found to correlate with increase in suppressor of cytokine signaling 3 (SOCS3) expression, which could be a possible explanation for immunosuppression. These observations demonstrate that after severe liver pathology, host derived factors play a dominant role in regulating LCMV specific antiviral immune immunity.

## Results

### Bile duct ligation results in persistence of virus following infection

**Figure 1:**
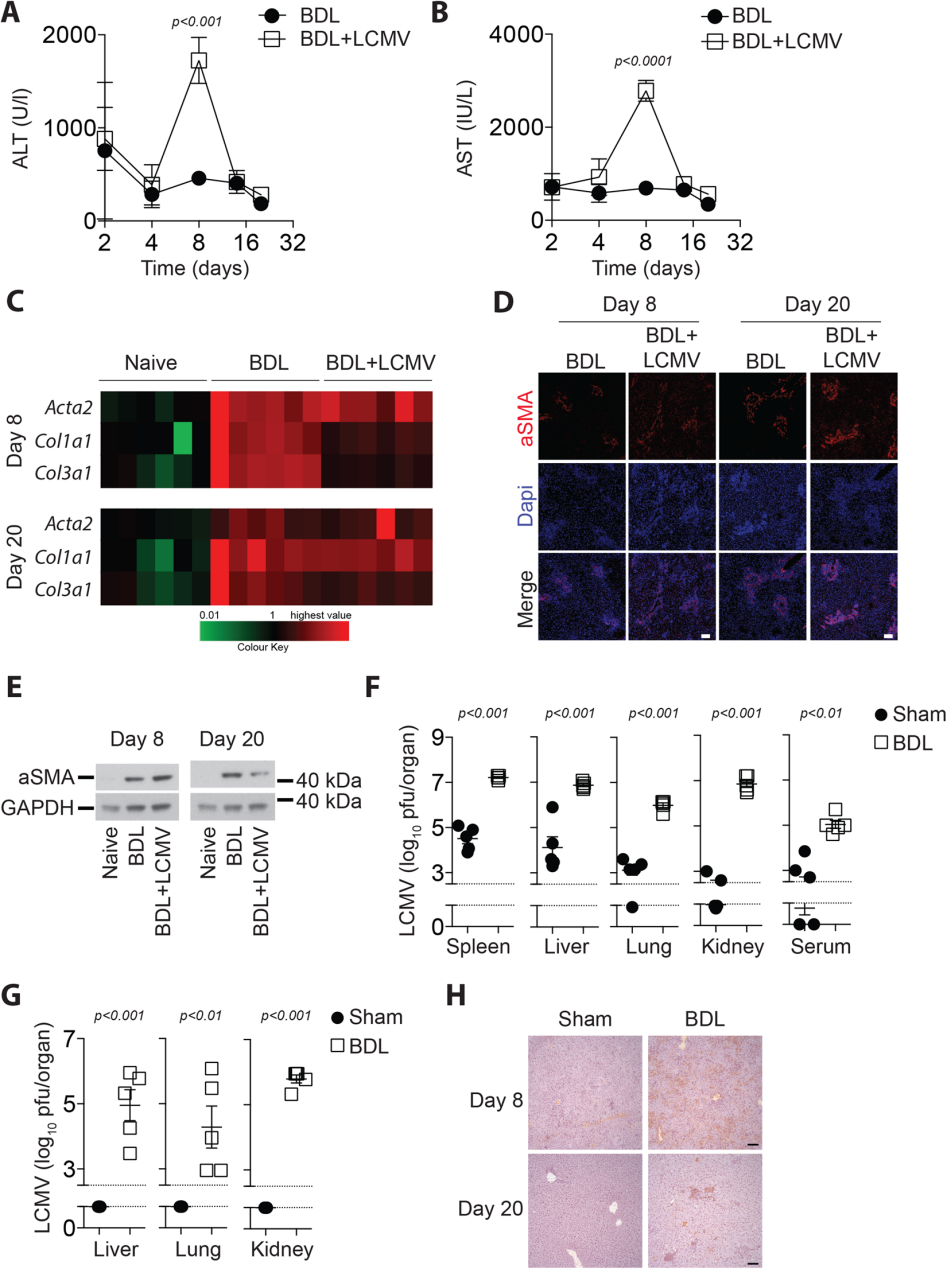
Bile duct ligation results in chronic viral infection. (**A–E**) Bile duct ligation was performed in C57Bl/6 mice. One day after the operation mice were infected with 2 × 10^4^ pfu of LCMV WE. (**A**) ALT activity and (**B**) AST activity was measured over time in infected and uninfected animals (n = 6). (**C**) *Acta2*, *Col1a1*, and *Col3a* RNA expression levels were determined 8 (upper panel) and 20 (lower panel) days after infection in infected or uninfected BDL treated mice (n = 6). (**D**) Sections from snap frozen liver tissue harvested from infected and uninfected BDL mice 8 days (left panels) and 20 days (right panels) p.i. were stained for α-SMA (red) and Dapi (blue). (One representative of n = 4 is shown, scale bar = 100 µm.) (**E**) Protein samples were isolated from liver tissue of naïve and uninfected or infected BDL mice following 8 days (left panels) and 20 days (right panels) p.i. α-SMA expression (upper panels) and GAPDH expression (lower panels) were determined (one representative immunoblot of n = 6 is shown). Uncropped western blot images are shown in Supplementary Fig. 8a. (**F**–**H**) Sham and BDL treated mice were infected with 2 × 10^4^ pfu of LCMV WE. Virus titers were measured in different organs as indicated (**F**) 8 days or (**G**) 20 days after infection (n = 5–7). (**H**) Sections from snap frozen liver tissue harvested from BDL (right panels) and Sham (left panels) treated animals 8 days (upper panels) and 20 days (lower panels) after infection and stained for LCMV-NP antibodies (Clone: VL4). Scale bar = 100 µm. One representative of n = 4–6 is shown).

Bile duct ligation in mice causes cholestasis and constant liver damage detectable by the presence of bilirubin and hepatic enzyme alanine aminotransferase (ALT) and aspartate aminotransferase (AST) levels in the blood stream (Supplementary Fig. 1A, Fig. 1A,B). When mice were infected with the non-cytolytic lymphocytic choriomeningitis virus (LCMV) following bile duct ligation (BDL), increases in liver damage markers were only significant at day 8 post infection (p.i.) relative to the BDL controls (Fig. 1A,B). With this LCMV dose we did not observe increased liver damage in Sham operated mice (Supplementary Figure 1C,D) but infection of sham operated mice led to upregulation of the liver fibrosis marker α-SMA and collagen (Supplementary Figure 1E,F). However, tissue inhibitor of metalloproteinases-1 (TIMP-1), a surrogate marker for liver fibrosis, was comparable in the serum of LCMV-infected and uninfected BDL animals (Supplementary Figure 1B). Consistently, α-SMA was similarly detected in liver tissue harvested from infected or control animals after BDL (Supplementary Figure 1H). Expression levels of collagens in liver tissue from BDL infected animals were similar to BDL controls 20 days after infection (Fig. 1C). α-SMA protein expression was comparable between BDL treated animals in the presence or absence of LCMV (Fig. 1D,E). These data suggest that LCMV infection did not alter the establishment of liver fibrosis following BDL. In this early setting after BDL, we did not observe bacteria in the spleen, liver or serum (Supplementary Figure 1I). However, there was a slight increase in *Ifnb* expression 2 days after BDL which is consistent with published data (Supplementary Figure 1J)^17^. Next, we wondered whether bile duct ligation affected the outcome of the viral infection. Interestingly, there were higher levels of LCMV in the organs of BDL animals compared to Sham operated controls 8 days following infection (Fig. 1F). Consequently, BDL treated animals developed a chronic viral infection with LCMV being detectable 20 days after infection in the BDL animals, while control animals eliminated the virus (Fig. 1G). LCMV infected cells were found in liver tissue in BDL mice, while no virus replication was observed in liver tissue of control animals (Fig. 1H). Taken together, these findings indicate that while LCMV infection had little impact on the development of liver fibrosis following BDL, a chronic viral infection was established in BDL animals following infection.

### Anti-viral T cell immunity is defective following bile duct ligation

**Figure 2:**
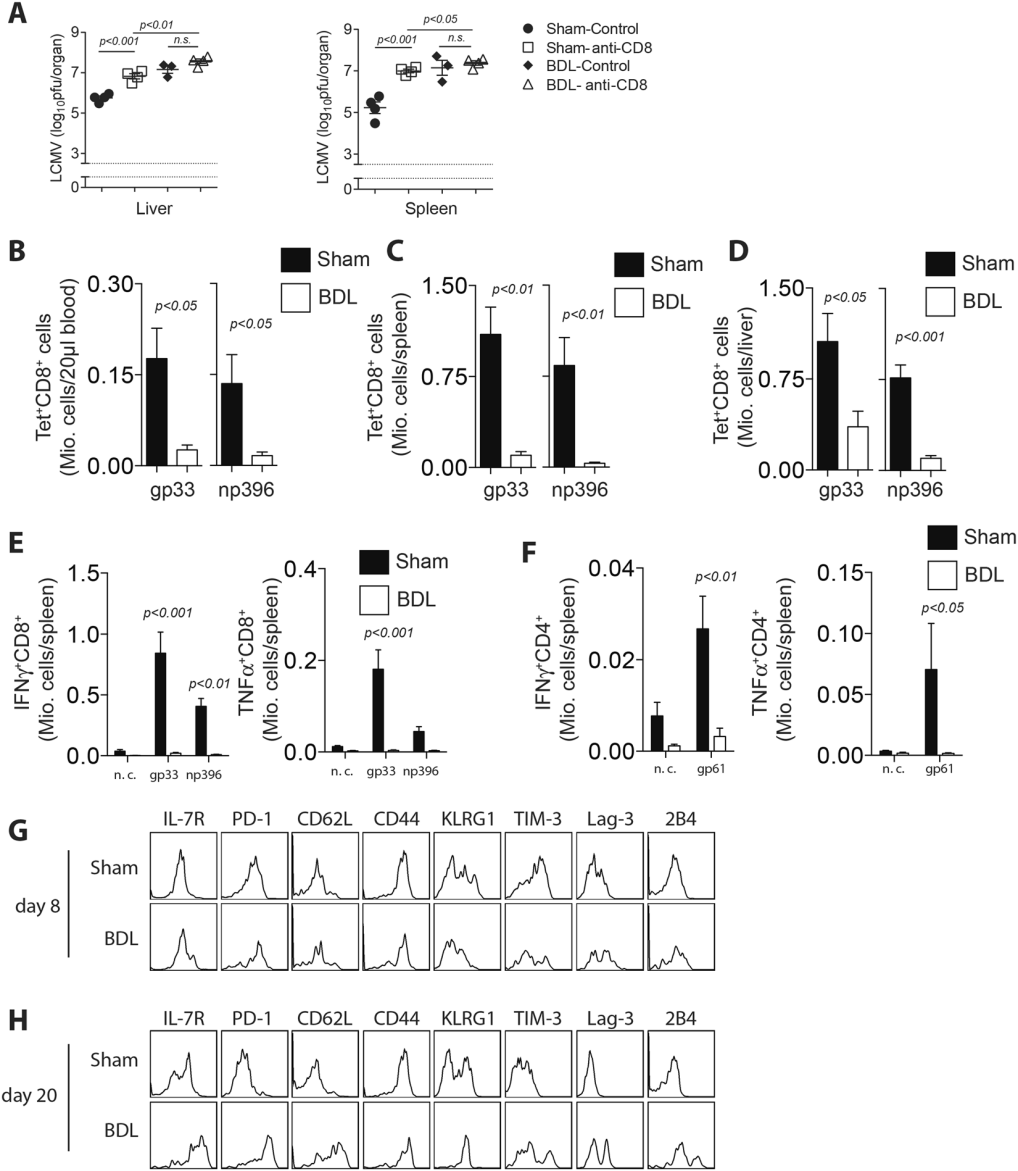
Anti-viral T cell immunity is defective following BDL. (**A**) CD8^+^ T cells were depleted in Sham and BDL mice and infected with LCMV WE. Virus titers were determined in spleen and liver 8 days after infection (n = 3–4). (**B–H**) C57Bl/6 mice were either operated with BDL or Sham following infection with 2 × 10^4^ pfu of LCMV WE. Virus specific T cells against gp33 (left panel) and np396 (right panel) were measured in (**B**) the blood, (**C**) spleen, or (**D**) liver tissue 8 days after infection (n = 7–8). (**E**) 8 days after infection, single cell suspended splenocytes were re-stimulated with the LCMV specific epitopes gp33 and np396. IFN-γ (left panel) and TNF-α (right panel) production was determined in CD8^+^ T cells. (**F**) 8 days after infection, single cell suspended splenocytes were exposed to the MHC-II epitope gp61. IFN-γ (left panel) and TNF-α (right panel) production was determined in CD4^+^ T cells. (**G**,**H**) Surface molecule expression was measured as indicated on gp33-H2-D^b^-tet^+^CD8^+^ T cells (**G**) 8 days, (one representative of n = 5 is shown) or (**H**) 20 days after infection. (One representative of n = 3–5 is shown).

Defense against LCMV infection requires effective virus specific T cells. Consistently, depletion of CD8^+^ T cells resulted in increased LCMV titers, which were comparable to LCMV titers in BDL operated mice (Fig. 2A, Supplementary Fig. 3A). Next we investigated whether BDL animals were able to mount an LCMV specific T cell response. As expected, virus specific T cells in the blood stream were highly reduced in bile duct ligated animals when compared to Sham controls 8 days p.i. (Fig. 2B). Consistently, LCMV specific T cells in spleen and liver tissue were decreased compared to Sham controls 8 days p.i. (Fig. 2C,D). LCMV specific T cells were also highly reduced in the spleen tissue of BDL treated mice 20 days following infection when compared to Sham controls, although there was no difference in liver tissue (Supplementary Figure 2A). Next, we investigated the cytokine production of anti-viral T cells. Following re-stimulation with LCMV specific epitopes, CD8^+^ T cells from Sham animals had higher IFN-γ and TNF production when compared to BDL animals (Fig. 2E). Similarly, CD4^+^ T cell IFN-γ and TNF production was also lower in BDL mice than in Sham controls (Fig. 2F). Analyses from liver tissue similarly showed defective T cell immunity 8 days (Supplementary Figure 2B,C) and 20 days p.i. (Supplementary Figure 2D–G). Loss of T cell immunity can be triggered by several factors which contribute to chronic viral infection. Interestingly, we observed increased PD-1, TIM-3, 2B4, and Lag-3 expression on LCMV specific T cells harvested from BDL treated animals when compared to Sham controls (Fig. 2G,H). This increased expression at day 20 could be a consequence of defective anti-viral immunity and viral persistence. NK cells can target anti-viral T cells and thus contribute to the establishment of a chronic viral infection^29–31^. We therefore wondered whether NK cell activation following BDL might target anti-viral T cells. However, following NK cell depletion, we still observed highly increased LCMV titers in several organs, arguing against a critical role of NK cells in this setting (Supplementary Figure 3B). Taken together, these data indicate that BDL limited anti-viral T cell immunity following LCMV infection.

### Serum compounds contribute to defective innate and adaptive immunity

**Figure 3:**
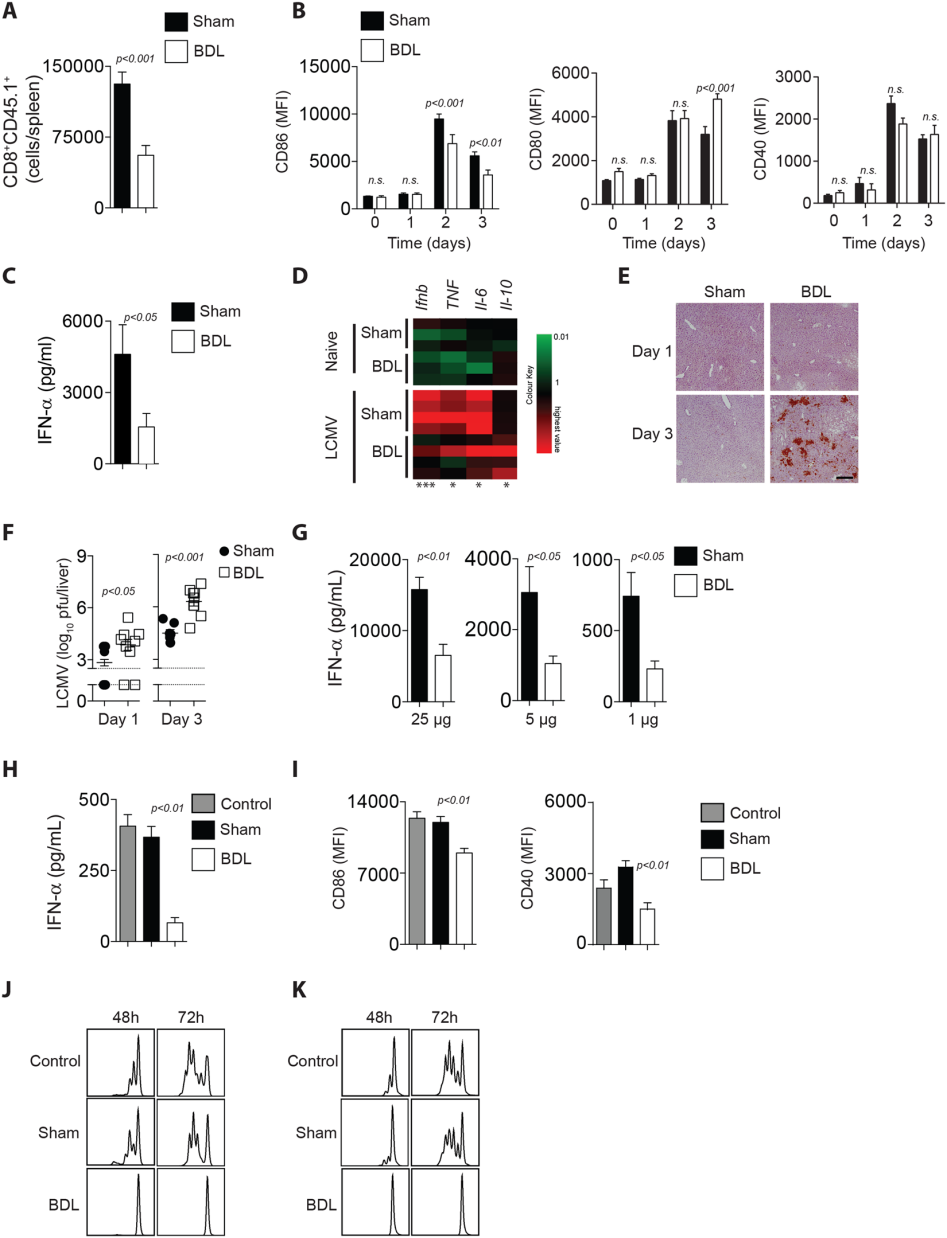
Serum factors contribute to defective immune activation following BDL. (**A**) C57Bl/6 mice were either operated with BDL or Sham followed by infection with 2 × 10^2^ pfu of LCMV WE. 2 × 10^6^ negatively sorted CFSE labeled CD8^+^ T cells from P14^+^ animals were transferred at day 2 p.i. At day 4 p.i., the cell numbers of transferred T cells were determined in the spleen (n = 8). (**B**–**F**) C57Bl/6 mice were either operated with BDL or Sham followed by infection with 2 × 10^4^ pfu of LCMV WE. (**B**) CD86, CD80 and CD40 expression was measured as mean fluorescence intensity (MFI) on the cell surface of BMDCs at the indicated time points following infection (n = 6–7). (**C**) IFN-α was measured in the serum 1 day after infection (n = 8). (**D**) RNA expression levels of the indicated genes were measured in spleen of naïve and LCMV infected (1 day p.i.) animals following Sham and BDL operation (n = 3–4), (*p* < *0*.*01* = **p < *0*.*001* = ***p < *0*.*0001* = ****). (**E**) Sections from snap frozen liver tissue harvested from Sham (left panels) and BDL (right panels) treated mice at day 1 (upper panels) and day 3 (lower panels) following infection were stained with anti-LCMV-NP antibodies (clone: VL-4). (One representative of n = 4 is shown, scale bar: 100 µm.) (**F**) Viral titers were determined in liver tissue 1 day (left panel) and 3 days (right panel) after infection (n = 7–10). (**G**) C57Bl/6 mice were either operated with BDL or Sham, 24 h later followed by injection of 25 µg (left panel), 5 µg (middle panel), and 1 µg (right panel) of polyI:C. IFN-α concentrations were determined after 3 h in the serum (n = 6–9). (**H**–**K**) Serum was harvested from C57Bl/6 mice 24 h after either BDL or Sham operation. (**H**,**I**) Bone marrow derived dendritic cells (BMDCs) were stimulated with polyI:C (50 µg/mL) in absence (control) and presence of serum of Sham and BDL treated animals. (**H**) IFN-α concentrations were determined after 24 h in the supernatant (n = 6). (**I**) CD86 and CD40 expression was determined on the cell surface of BMDCs 24 h following stimulation (n = 7). (**J**) 2 × 10^6^ negatively sorted CFSE labeled CD8^+^ T cells from P14^+^ animals were co-incubated with gp33 pulsed BMDCs in the absence (control) and presence of serum harvested from Sham or BDL operated animals. After 24 h, CFSE fluorescence was determined on T cells (one representative set of histograms of n = 6 is shown). (**K**) Negatively sorted CFSE labeled CD8^+^ T cells were stimulated with anti-CD3 and anti-CD28 antibodies in the absence (control) and presence of serum harvested from Sham or BDL operated animals. CFSE abundance was measured at the indicated time points (one representative of n = 6 is shown).

Next we wondered whether defective T cell immunity resulted from reduced T cell activation following infection. Accordingly, we transferred negatively sorted carboxyfluorescein succinimidyl ester (CFSE) labeled T cells from mice expressing an LCMV-gp33 specific TCR as a transgene (P14) into LCMV infected BDL or Sham mice. We observed a reduced presence of transferred T cells in BDL treated animals as compared to Sham mice (Fig. 3A). T cell activation is mediated by antigen presenting cells including CD11c^+^MHC-II^+^ dendritic cells^32^ but, we did not observe differences in numbers of CD11c^+^MHC-II^+^ cells in BDL mice when compared to Sham controls (Supplementary Figure 4A–C). However, we observed reduced expression of the co-stimulatory molecule CD86 on day 2 and 3 p.i. Expression of CD80 was increased 3 days p.i. and CD40 expression was not significantly different on dendritic cells in BDL mice when compared to control mice following infection (Fig. 3B). When we measured the IFN-α concentration in the serum of infected mice, the IFN-α levels were higher in Sham mice compared to their BDL counterparts (Fig. 3C). We also observed reduced RNA expression levels of pro-inflammatory cytokines in BDL mice compared to Sham controls after infection (Fig. 3D). As the numbers of plasmacytoid dendritic cells were not reduced in spleen tissue harvested from BDL mice compared to Sham mice (Supplementary Figure 4D–F), we speculated that cholestasis or the consequent liver damage might induce regulatory mechanisms to block cytokine production following pathogen recognition receptor (PRR) stimulation. Consequently, we observed increased virus replication in BDL mice following infection in the liver (Fig. 3E,F). When we stimulated mice 1 day after BDL or Sham operation with the TLR3 agonist polyI:C, we observed reduced IFN-α production in BDL mice when compared to Sham animals (Fig. 3G). As IFN-α production following polyI:C injection most likely requires presence of DCs in the spleen, we hypothesized that the compound interfering with PRR stimulation may be present in the blood stream. When we incubated serum harvested from mice 24 h after BDL or the Sham operation with bone marrow derived DCs (BMDCs) followed by stimulation with polyI:C, we observed lower IFN-α production in the presence of BDL (Fig. 3H). Additionally, upregulation of CD86 and CD40 on BMDCs by polyI:C was reduced in the presence of BDL serum when compared to the presence of Sham serum (Fig. 3I). Next, we wondered whether induction of T cell proliferation by BMDCs was reduced in the presence of BDL serum. We therefore exposed negatively sorted, CFSE labeled T cells from P14^+^ splenocytes to gp33 and pulsed BMDCs in the presence of BDL or Sham serum. Interestingly there was no difference between control (no serum) and Sham serum (Fig. 3J). However, in presence of BDL serum T cell proliferation was highly reduced (Fig. 3J). When we stimulated T cells using anti-CD3, anti-CD28 we obtained similar results (Fig. 3K). Taken together, these data indicate that serum compounds are able to inhibit cytokine production in BMDCs as well as T cell proliferation.

### Several serum factors including bilirubin and grp78 are upregulated following cholestasis

**Figure 4:**
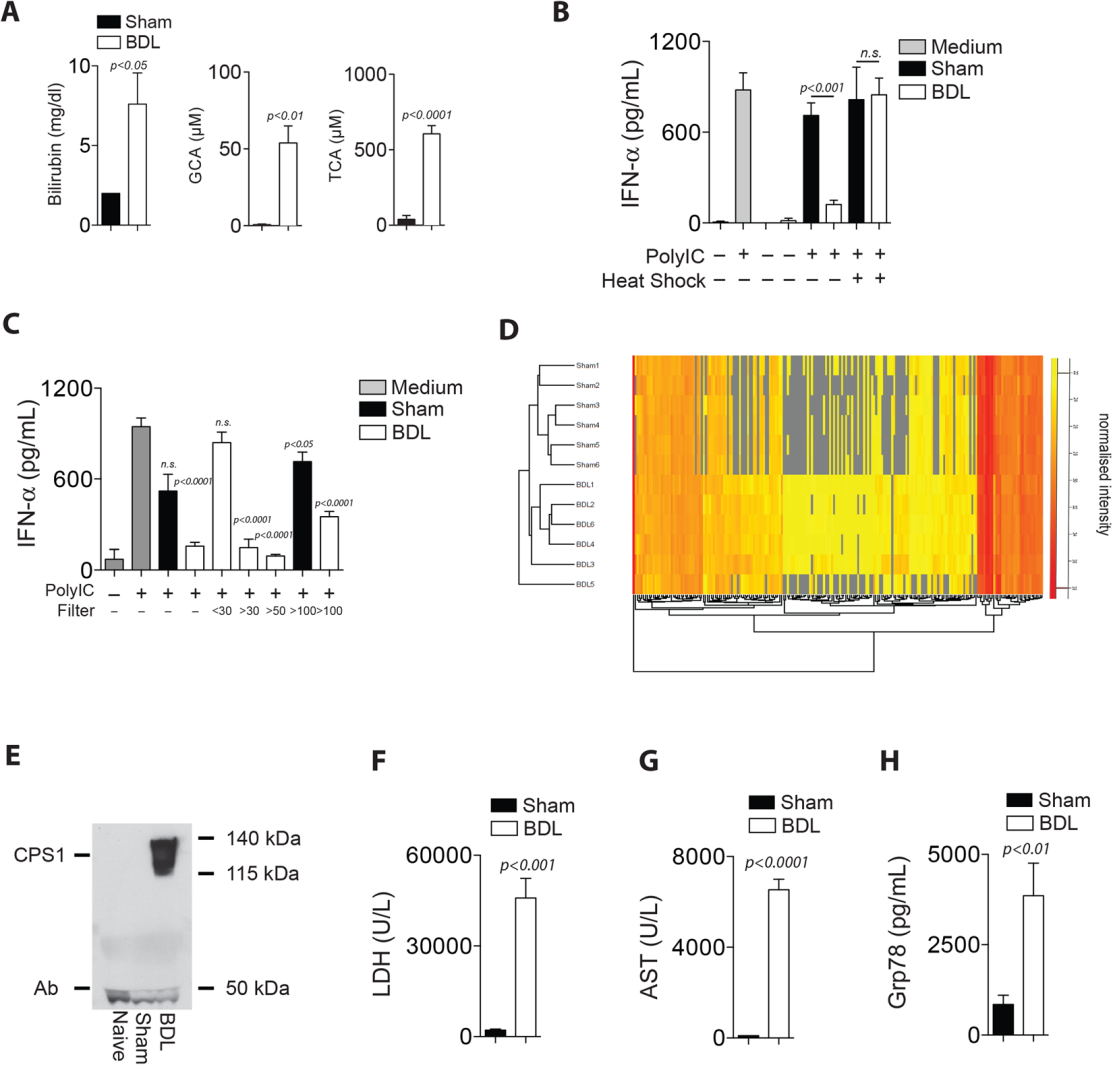
Fractionation and mass spectrometry analyses uncover several factors including GRP78 to be increased in the serum following BDL. (**A**–**H**) Serum was harvested from C57Bl/6 mice 24 h after either BDL or Sham operation. (**A**) Bilirubin, GCA and TCA were measured in serum from C57Bl/6 mice 24 h after either BDL or Sham operation (n = 6). (**B**) Bone marrow derived dendritic cells (BMDCs) were stimulated with polyI:C (50 µg/mL) in the absence and presence of serum from Sham or BDL treated animals before and after heat inactivation (n = 3). (**C**) Serum proteins from Sham or BDL treated animals were separated according to their protein size using spin columns or dialysis bags as indicated. Next, BMDCs were stimulated with polyI:C (50 µg/mL) in the absence and presence of the serum fractions and the IFN-α concentration was measured 24 h later (n = 3–18). (**D**) Serum proteins were separated using a 100 kDa dialysis bag followed by protein digestion and quantitative mass spectrometry. The normalized signal intensity of identified proteins from Sham and BDL samples (n = 6) is shown in a hierarchical cluster analysis. (**E**) Immunoblot of whole serum samples harvested from naïve, Sham or BDL animals following immunostaining with anti-CPS-1 (carbamoyl phosphate synthetase) and an anti-mouse secondary antibody is shown (one representative of n = 4 is shown). Uncropped western blot images are provided in Supplementary Fig. 8b. (**F**) LDH activity and (**G**) AST activity were measured in serum from Sham and BDL operated mice (n = 5). (**H**) GRP78 was measured in the serum from Sham and BDL operated mice (n = 7).

Next, we wondered which serum components in BDL mice were able to inhibit BMDC stimulation and T cell proliferation. BDL results in cholestasis and as a result, a variety of bile components, including bilirubin, GCA and TCA could be detected in the blood stream following BDL (Fig. 4A, Supplementary Table S1). Following heat shock treatment, BDL serum lost the ability to inhibit IFN-α production in polyI:C treated BMDCs (Fig. 4B). Hence, next we chose to determine which proteins contribute to suppressing immune activation. Specifically, we filtered BDL serum using multi-sized filters. The inhibitory effect of BDL serum was still observed in the protein filtrate >100 kDa (Fig. 4C). Using mass spectrometry analyses we found 66 proteins showing a higher abundance in the 100 kDa fraction in BDL serum compared to Sham serum (Fig. 4D, Supplementary Table S2). As expected, enzymes which are predominantly located in the liver such as Carbamoyl phosphate synthetase (CPS1), lactate dehydrogenase (LDH), AST, and ALT were detected in the serum of BDL animals when compared to Sham controls (Fig. 4E–G, Supplementary Table S2, Supplementary Figure 5A). Among the identified proteins, we observed GRP78/BIP to be significantly increased in the serum of BDL mice when compared to Sham controls (Fig. 4H). Taken together, these data indicate that several factors including bilirubin and GRP78/BiP were upregulated in the serum harvested from BDL treated mice as compared to Sham controls.

### Bilirubin and grp78 inhibit immune activation

**Figure 5:**
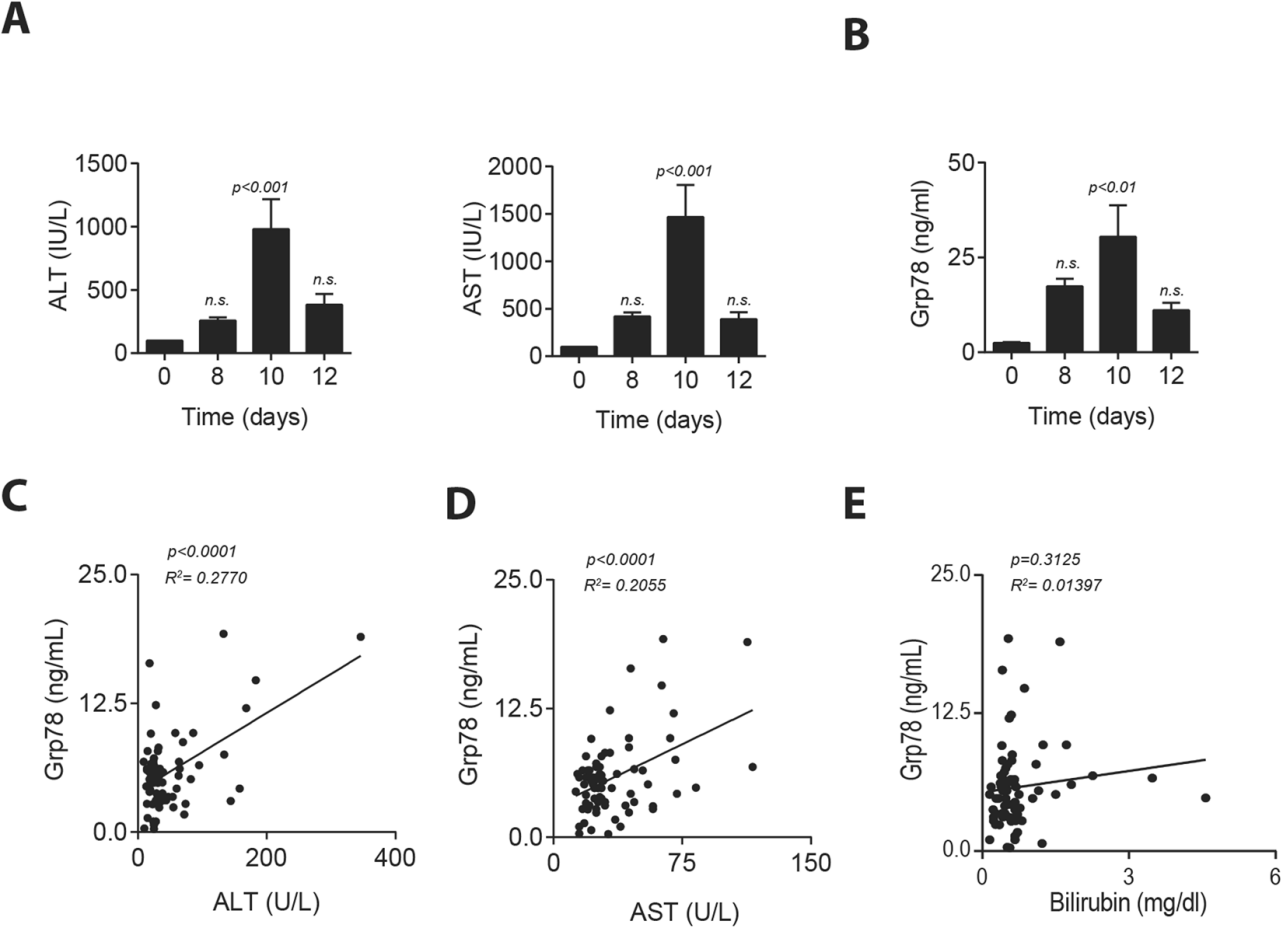
Liver damage triggers increase of GRP78 serum concentration. (**A**,**B**) WT mice were infected with 2 × 10^6^ PFU of LCMV WE strain without BDL and (**A**) ALT, AST levels were determined in the infected mice at day 8, 10 and 12 post infection (n = 8). (**B**) GRP78 was measured in the serum (n = 3–9) (**C–E**) Human GRP78 levels in patient serum is represented as a function of (**C**) ALT, (**D**) AST and (**E**) bilirubin from the patient cohort described in Supplementary Table 3 (n = 77).

Next, we wondered whether GRP78 is only present during BDL or also other conditions. High doses of LCMV WE can induce CD8^+^ T cell mediated liver damage in absence of BDL or other treatments^33,34^. As expected, ALT and AST activity in the sera of LCMV infected mice without surgery was increased (Fig. 5A). The concentration of GRP78 in this time frame was also increased (Fig. 5B). Next, we exposed human hepatocytes (HepG2 cells) to bile acids and did not observe an increase in ALT activity while destruction of cell integrity resulted in increased ALT activity in the lysate (Supplementary Figure 6A). Consistently, we found increased GRP78 concentrations in the lysates of hepatocytes but not the supernatant of cells treated with bile acids (Supplementary Figure 6B). These data indicate that GRP78 is likely to be released during destruction of hepatocytes rather than actively secreted following liver damage or exposure to bile acids. Hence, we wondered whether increase of GRP78 can be also found in human patient samples during liver damage (Supplementary Table S3). Interestingly, increased ALT and AST activity correlated with serum concentrations of GRP78 in human patient samples (Fig. 5C,D). Notably, we found no significant correlation between bilirubin and GRP78, indicating that both factors are present independently in patient samples (Fig. 5E). Taken together, our data suggests that release of GRP78 can occur through hepatocyte death during liver pathology.

**Figure 6:**
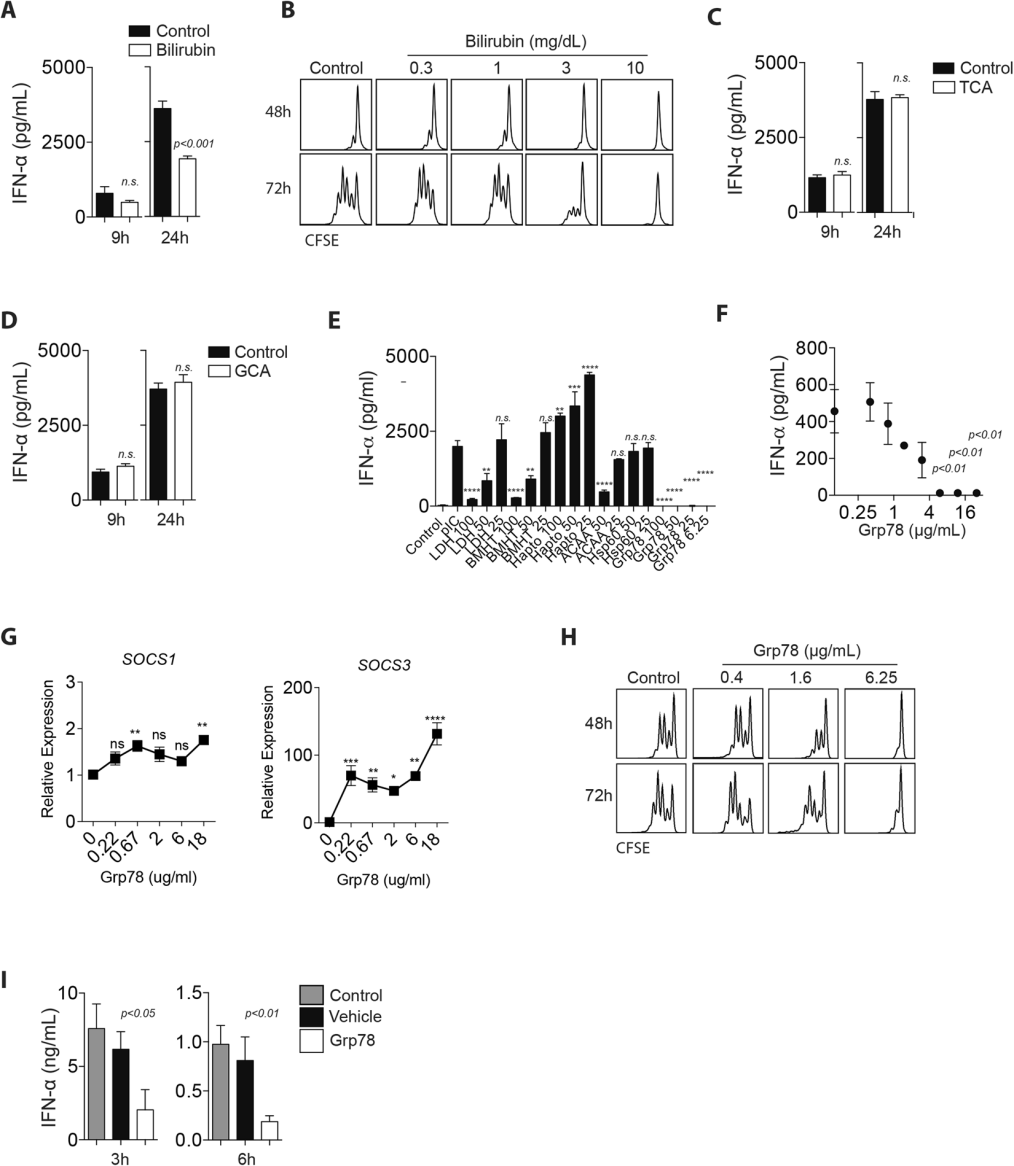
Several factors trigger defective immunity in BDL serum. (**A**) BMDCs were stimulated with polyI:C (50 µg/mL) in absence and presence of 60 mg/dl bilirubin. IFN-α concentration was measured 9 h and 24 h later (n = 4). (**B**) Negatively sorted CFSE labeled CD8^+^ T cells were stimulated with anti-CD3 and anti-CD28 antibodies in absence (control) and presence of different bilirubin concentrations (as indicated). CFSE abundance was measured at indicated time points (one representative of n = 6 is shown). (**C-D**) BMDCs were stimulated with polyI:C (50 µg/mL) in absence and presence of either (**C**) TCA, or (**D**) GCA. IFN-α concentration was measured 9 h and 24 h later (n = 4). (**E**) BMDCs were stimulated with polyI:C (50 µg/mL) in absence and presence of different concentrations of recombinant proteins as indicated. IFN-α concentration was measured 24 h later (n = 3). (*p* < *0*.*01* = **p < *0*.*001* = ***p < *0*.*0001* = ****) (**F**) BMDCs were exposed to a wide range of GRP78 concentrations during stimulation with polyI:C (50 µg/mL). IFN-α concentrations were determined in the supernatant (n = 3). (**G**) BMDCs were stimulated with GRP78 at indicated concentration for 24 h, mRNA was isolated and evaluated for SOCS1 and SOCS3 gene expression (n = 4). (**H**) Negatively sorted CFSE labeled CD8^+^ T cells were stimulated with anti-CD3 and anti-CD28 antibodies in the absence (control) and presence of different GRP78 concentrations. CFSE abundance was measured at indicated time points (one representative of n = 3 is shown). (**I**) GRP78 (25 ug) or vehicle was administered to mice at day -1 and day 0 following injection with polyI:C (25 µg) in these and untreated (control) mice. After 3 h and 6 h, IFN-α concentrations were determined in the serum (n = 4–7).

Next, we wondered whether bilirubin or bile acids can contribute to defective immune activation. When we stimulated BMDCs with polyI:C in the presence of bilirubin (60 mg/dl), we observed only a modest reduction in IFN-α production (Fig. 6A). Bilirubin inhibited T cell proliferation at high concentrations (Fig. 6B), which is consistent with the literature^35^. The most prominent change amongst the bile acids was observed in glycocholic acid (GCA) and taurocholic acid (TCA) (Supplementary Table 1). However, when we stimulated BMDCs with polyIC in presence of TCA or GCA, we observed no differences in IFN-α production (Fig. 6C,D).

Next, we wondered whether GRP78 exhibits regulatory effects during immune activation, following reports describing its beneficial role during rheumatoid arthritis^36^. Interestingly, GRP78 showed a potent inhibition of BMDC derived IFN-α production, while other proteins identified by mass spectrometry showed little or weak effects (Fig. 6E). GRP78 deficient mice are lethal and conditional hepatic GRP78 deficient animals show only a reduction in GRP78 expression and exhibit steatohepatitis due to the important intracellular functions of GRP78^15,16^. Following incubation with different concentrations of human recombinant GRP78, IFN-α production was inhibited in a concentration dependent manner (Fig. 6F). Incubation with GRP78 induced suppressor of cytokine signaling 1 and 3 (SOCS1 and 3) (Fig. 6G). However, GRP78 did not induce cell death in BMDC cultures (Supplementary Figure 7A). Furthermore, T cell proliferation was reduced in presence of GRP78 when compared to cultures in the absence of GRP78 (Fig. 6H). These data indicate that GRP78 can inhibit immune activation *in vitro*. Application of the ER stress agent tunicamycin can limit adaptive immunity following LCMV infection^37^. However, we did not detect increased liver enzymes or increased GRP78 concentration in the sera following tunicamycin injection (Supplementary Figure 7B,C). Moreover, we did not observe a difference in IFN-I production in the absence or presence of tunicamycin (Supplementary Figure 7D). When we injected GRP78 intra peritoneally at day -1 and day 0 we measured an increase of GRP78 concentrations in the serum (Supplementary Figure 7E). Interestingly, following injection of GRP78 and polyI:C, we observed reduced IFN-α concentration in GRP78 treated animals when compared to controls (Fig. 6I). Taken together, these data indicate that bilirubin and GRP78 can inhibit innate immune activation *in vitro* and *in vivo*.

## Discussion

In this study, we demonstrated that bile duct ligation can cause immune suppression during LCMV infection resulting in chronic viral infection. We observed reduced T cell immunity and IFN-I production following LCMV infection. Mass spectrometric analysis of serum samples after cholestasis revealed upregulation of various proteins such as Lactate dehydrogenase (LDH), Betaine—homocysteine S-methyl transferase (BMHT), 3-Ketoacyl-CoA thiolase (ACAA2) and GRP78. Bilirubin and GRP78 suppressed IFN-I production and T cell proliferation *in vitro*.

Bacterial translocation results in prolonged IFN-I expression in the liver following BDL^17^. Both, IFN-I and IFN-II are critical factors that limit viral replication and contribute to clearance of a viral infection^20^. However, our data suggests that cholestasis and the consequent liver damage inhibits IFN induction following LCMV infection and TLR3 stimulation and accordingly drives excessive viral replication. Notably, IFN-I expression during liver fibrosis is usually observed at later time points, with associated microbial translocation and immune activation^17^. Consistently, IFN-I can also trigger regulatory effects on anti-viral T cells, which may contribute to limited anti-viral T cell immunity against LCMV^23,24^. Although in our settings we see limited IFN-I at early stages, microbial translocation could trigger IFN-I production and thus T cell dysfunction during later stages.

Since we observed increased viral titers at day 3 after infection, we speculated that defective innate immune activation contributes to viral persistence and defective T cell immunity. Since the immunoregulatory effects were reiterated in tissue culture experiments, we speculated that following cholestasis, serum components limit immune activation to circumvent lethal pathology. Moreover, redundant co-stimulatory signals during viral infection ensure productive and sufficient immune activation^38^.

In our setting, we observed slightly reduced expression of co-stimulatory molecules in addition to defective IFN-I production following LCMV infection. A recent study has reported that cholestasis can induce anti-inflammatory signals in mice via increased IL-10 production following BDL^39^. Notably, increased IL-10 production can be observed following exposure of CD8^+^ T cells towards GRP78^40^. While we also observed more RNA expression of *Il-10* following BDL, it remains unclear whether this effect is dependent on GRP78 expression. Moreover, increased presence of bilirubin can inhibit antigen specific T cell immunity^35^. Although individual proteins showed immunosuppressive phenotype *in vitro*, its detailed relevance *in vivo* needs further characterization. Furthermore, hepatocyte specific deletion of proteins detected by mass spectrometry would be useful in elucidating the role of individual proteins during cholestasis. We postulate that a combination of several of the above mentioned mechanisms orchestrate the strong immune suppression in our setting.

The unfolded protein response plays an important role during immune activation^41^. Specifically, the GRP78 binding receptor IRE-1α mediates activation of XBP-1 to control the homeostasis of CD8^+^ DCs^42^. Furthermore, TLR stimulation can result in activation of IRE-1α, even in the absence of ER stress^43–45^. In turn, ER stress can trigger inflammatory signals via NLRP3 and nucleotide-binding oligomerization domain-like receptors (NOD1, and NOD2)^46,47^. Viruses can modulate the unfolded protein response to promote viral replication^41^. The murine cytomegalovirus protein M50 triggers downregulation of IRE-1α and consequently XBP-1 signaling^48^. In turn, Japanese encephalitis virus activates UPR to target degradation of host cell proteins while leaving the viral proteins unaffected^49^.

Although the relevance of endogenous GRP78 remains to be established, this protein may act as an immune suppressor following tissue damage. Autoantibodies against GRP78 have been linked to rheumatoid arthritis and it is tempting to speculate that neutralization of GRP78 may promote autoimmunity^50^. GRP78 is highly expressed in cancer cells, and surface expression of GRP78 has been linked to carcinogenesis^51,52^. Considering our data, GRP78 may act as an inhibitor of anti-tumor immunity and consequently protect cancer cells. In turn, blocking of surface GRP78 may increase specific anti-tumor defense mechanisms and provide an effective anti-tumor therapeutic strategy.

In conclusion, we found that cholestasis mediated liver damage results in defective immune activation leading to persistent LCMV infection.

## Materials and Methods

### Mice, viruses, virus titration

Experiments were performed in 12 weeks old mice. All animal experiments were approved by institutional ethics committee at the Landesamt für Natur, Umwelt und Verbraucherschutz of North Rhine-Westphalia (LANUV NRW), Germany in accordance with the German laws for animal protection. All experiments were performed in accordance with institutional guidelines and regulations. Use of human samples were approved by internal ethics committee of the Faculty of Medicine at the Heinrich-Heine-University of Düsseldorf under the Study-No. 5350. Informed consent was obtained from the patients. For bile duct ligation, animals were anaesthetized by isoflurane and placed on a heating pad. After intubation and ventilation (Harvard minivent, Harvard apparatus, Holliston, USA), the animals were shaved and the skin disinfected with 70% ethanol and povidone-iodine. A midline incision in the upper abdomen was made; the common bile duct and the bile bladder were identified, isolated and ligated with silk. The fascia and skin of the midline abdominal incision were closed with silk. Sham treatment was performed similarly but without ligation of the bile duct and bile bladder. Animals were monitored until recovery and treated with carprofen (0.05 mg/kg b.w.) after the procedure. NK cells were depleted with intravenous (i.v.) injections of anti-NK1.1 antibody (clone PK136) as previously described^30,53^. LCMV strain WE was originally obtained from F. Lehmann-Grube (Heinrich Pette Institute, Hamburg, Germany) and was propagated in L929. Mice were infected with virus via tail vein injection. Virus titers were measured using a plaque forming assay as previously described^54,55^. Mice were administered with two doses of Grp78 (25 μg/mouse) (Biomatik) 24 h apart and one dose of 25 μg/mouse of poly I:C (GE healthcare life sciences). Tunicamycin (Sigma) from *Streptomyces* sp. was injected intravenously as previously described^37^.

### Cell Culture

HepG2 cells were obtained from ATCC. Cells were maintained in DMEM supplemented with 10% fetal calf serum (FCS), penicillin and streptomycin in. To obtain BMDCs, bone marrow was isolated from C57Bl/6 mice. Approximately 2 × 10^6^ cells were seeded on culture dishes with 10% FCS in RPMI medium with GM-CSF (40 ng/ml) for 10 days. Cells were harvested on day 10 and treated with GRP78 (BioMatik) and poly I:C (50 μg/ml) (Sigma). Apoptosis was assessed using Annexin V and 7AAD staining (Thermofisher). HepG2 cells were treated with glycocholic Acid (GCA), glycochenodeoxycholic acid (GCDC), taurocholic acid (TCA) and taurochenodeoxycholate (TCDC) (All from Sigma) indicated concentrations.

### Purification of T cells

For T cell purification, single cell suspended splenocytes were enriched following the manufacturer’s instructions using the pan T cell MACS kit and the CD8 purification kit (Miltenyi, Germany). Purified T cells were labelled with CFSE as previously described (Invitrogen)^30^.

### Flow cytometry analysis

Flow cytometry and tetramer were performed as previously described^55^. Briefly, single cell suspended splenocytes were incubated with gp33-tetramer and np396-tetramer for 15 minutes and gp-66 tetramer for 30 minutes at 37 °C. After the incubation, surface antibodies were added, including anti-CD8 or anti-CD4 along with anti-IL7R, anti-PD-1, anti-CD62L, anti-CD44, anti-KLRG-1, anti-TIM3, anti-Lag-3, and anti-2B4 antibodies (eBioscience) for 30 minutes at 4 °C. For intracellular cytokine stain single suspended splenocytes were incubated with the LCMV specific peptides gp33 and np396 or the MHC-II epitope gp61. After 1 h, Brefeldin A (eBioscience) was added, followed by an additional 5 h incubation at 37 °C. After surface stain with anti-CD8 or anti-CD4 antibodies (eBioscience) cells were fixed with 2% formalin and permeabilized with 0.1% Saponin and stained with anti-IFNγ and anti-TNF antibodies (eBioscience) for 30 min at 4 °C.

### RT-PCR analyses

RNA purification and RT-PCR analyses were performed according to manufacturer’s instructions (Qiagen). Gene expression analysis of *Acta2*, *Col1a1*, *Col3a*, *Ifnb*, *Tnfa*, *Il-10*, *Il-6*, *Socs1 and Socs3* was performed using kits from Applied Biosystems. For analysis, the expression levels of all target genes were normalized to *Gapdh* or *β-actin* expression (∆Ct). Gene expression values were then calculated based on the ∆∆Ct method, using the mean naive mice as a control to which all other samples were compared. Relative quantities (RQ) were determined using the equation: RQ = 2^−∆∆Ct.

### Histology and ELISA

Histological analysis on snap frozen tissue was performed as previously described^56^. Briefly, 7 μm liver tissue sections were fixed with acetone for 10 min, blocked with 10% FCS in phosphate buffer saline (PBS) for 1 h. Stained with anti-α-smooth muscle actin (SMA) (Abcam), LCMV-NP antibody (Clone: VL4) for 1 h. Sections were mounted with fluorescent mounting media (Dako) or Kaiser’s mounting media (Sigma). TNF, IFN-γ, IL10 (eBioscience), IFN-α ELISAs (PBL Biosciences) and GRP78 (LS bioscience, MyBioSource) were performed according to the manufacturer’s instructions.

### Serum preparations

For heat inactivation experiments, 100 µl of serum was mixed with 100 µl of PBS followed by heating at 95 °C for 10 minutes. For isolation of the 30 kDa fraction, 500 µl of serum was separated using a 30 kDa Amikon Ultra centrifugal filter (Millipore). For isolation of the 50 kDa or 100 kDa fractions, 500 µl serum were dialyzed using a 50 kDa or 100 kDa Float-A-Lyzer G2 dialysis device (Spectrum laboratories) for 24 h against 3 l of PBS 3 times at 4 °C.

### Immunoblotting

Whole cell lysate were prepared using 1% TritonX in phosphate buffer saline. Immunoblots were probed with primary alpha-SMA (alpha smooth muscle actin) and anti-CPS1 (Carbamoyl phosphate synthetase) and developed using anti-rabbit HRP.

### Mass spectrometry

5 µg of serum samples from six individual BDL and six individual sham mice were analyzed by liquid chromatography coupled electrospray ionization tandem mass spectrometry. The samples were separated over a 4 mm running distance in a 4–12% polyacrylamide bis-tris gel (Life Technologies, Darmstadt, Germany). After silver staining, protein containing bands were cut out, destained, reduced and alkylated as described^57^. After tryptic digestion with 0.1 µg trypsin (Serva, Heidelberg, Germany) in 50 mM ammonium hydrogen carbonate overnight, peptides were extracted from the gel with 1:1 (v/v) 0.1% trifluoroacetic acid / acetonitrile and after removal of acetonitrile ~ 500 ng peptides subjected to liquid chromatography in 0.1% trifluoroacetic acid.

Peptides were separated using an Ultimate 3000 Rapid Separation liquid chromatography system (Thermo Scientific, Dreieich, Germany). First, peptides were pre-concentrated on an Acclaim PepMap100 trap column (3 µm C18 particle size, 100 Å pore size, 75 µm inner diameter, 2 cm length, Thermo Scientific, Dreieich, Germany) at a flow rate of 6 µl / min using 0.1% (v/v) TFA as mobile phase. After 10 min, peptides were separated on an analytical column (Acclaim PepMapRSLC, 2 µm C18 particle size, 100 Å pore size, 75 µm inner diameter, 25 cm length, Thermo Scientific, Dreieich, Germany) at 60 °C by a 2 h gradient from 4 to 40% solvent B (solvent A: 0.1% (v/v) formic acid in water, solvent B: 0.1% (v/v) formic acid, 84% (v/v) acetonitrile in water) at a flow rate of 300 nl/min.

Mass spectrometric analysis was carried out on a Q Exactive plus (Thermo Scientific, Bremen, Germany) quadrupole-orbitrap mass spectrometer. Full scans were recorded in the orbitrap analyser in profile mode with a resolution of 70000 over a scan range from 350 to 2000 m/z with a maximum ion time of 80 ms and the targed value for the automatic gain control set to 3000000. Up to ten two- and threefold charged precursor ions were isolated by the build in quadrupole within a 2 m/z isolation window, fragmented via higher-energy collisional dissociation and fragments analysed in the orbitrap analyser with a maximal ion time of 60 ms and the targed value for the automatic gain control set to 100000. The resolution was set to 17500 and the available scan range 200 to 2000 m/z and spectra were recorded in centroid mode. Already fragmented precursors were excluded from analysis for the next 100 seconds; the capillary temperature was set to 250 °C.

For protein and peptide identification and quantification the MaxQuant environment (version 1.5.3.8, MPI for biochemistry, Martinsried, Germany) was used applying default parameters if not otherwise stated. Spectra were searched against 16727 mouse entries form the SwissProt part of the UniProtKB (downloaded on 11th November 2016). A precursor mass tolerance of 20 ppm (4.5 ppm after recalibration) was allowed and 20 ppm for fragment masses. Furthermore, trypsin specific cleavage with a maximum of two missed cleavage sites was considered, carbamidomethyl as fixed and methionine oxidation and N-terminal acetylation as variable modifications. Peptide and protein identifications were accepted at a false discovery rate of 1% and only proteins identified with a minimum of two peptides were considered for further analysis within the Perseus environment (version 1.5.2.4, MPI for biochemistry, Martinsried, Germany). Here, contaminants were filtered out and only proteins considered showing in at least 5 samples of one group were considered valid values. Before statistical analysis by the significance analysis of microarray method (Welch’s t-tests, S_0_ = 0.1, false discovery rate 5%) on log2-transformed values, missing data was imputed with random values from a downshifted normal distribution (width: 0.3 SD, downshift 1.8 SD).

### Statistical analysis

Data are expressed as mean ± S.E.M. Statistical significance between two groups was analyzed using students t-test. For experiments involving analysis of multiple time points, two-way ANOVA with an additional Bonferroni post-test was used. Mantel-Cox test was used for analysis of survival curves. p-values < 0.05 were considered to be statistically significant.

## Electronic supplementary material

Supplementary information
