# Supplementary material for: Cholestasis induced liver pathology results in dysfunctional immune responses after arenavirus infection
Source: Sci Rep. 2018 Aug 15;8:12179. doi: 10.1038/s41598-018-30627-y (PMC6093869; doi:10.1038/s41598-018-30627-y)
Supplement: Supplementary file 1 — Supplementary information [file 41598_2018_30627_MOESM1_ESM.pdf]

Title: Cholestasis induced liver pathology results in dysfunctional immune responses after arenavirus infection

Elisabeth Lang[1, 2, #], Vitaly I. Pozdeev[2, #], Prashant V. Shinde[2, #], Haifeng C. Xu[2], Balamurugan Sundaram[2], Yuan Zhuang[2], Gereon Poschmann[3], Jun Huang[2], Kai Stühler[3, 5], Aleksandra A. Pandyra[1, 4], Verena Keitel[1], Dieter Häussinger[1], Karl S. Lang[4] and Philipp A. Lang[2, *]

Supplementary Info

Supplementary Figure 1

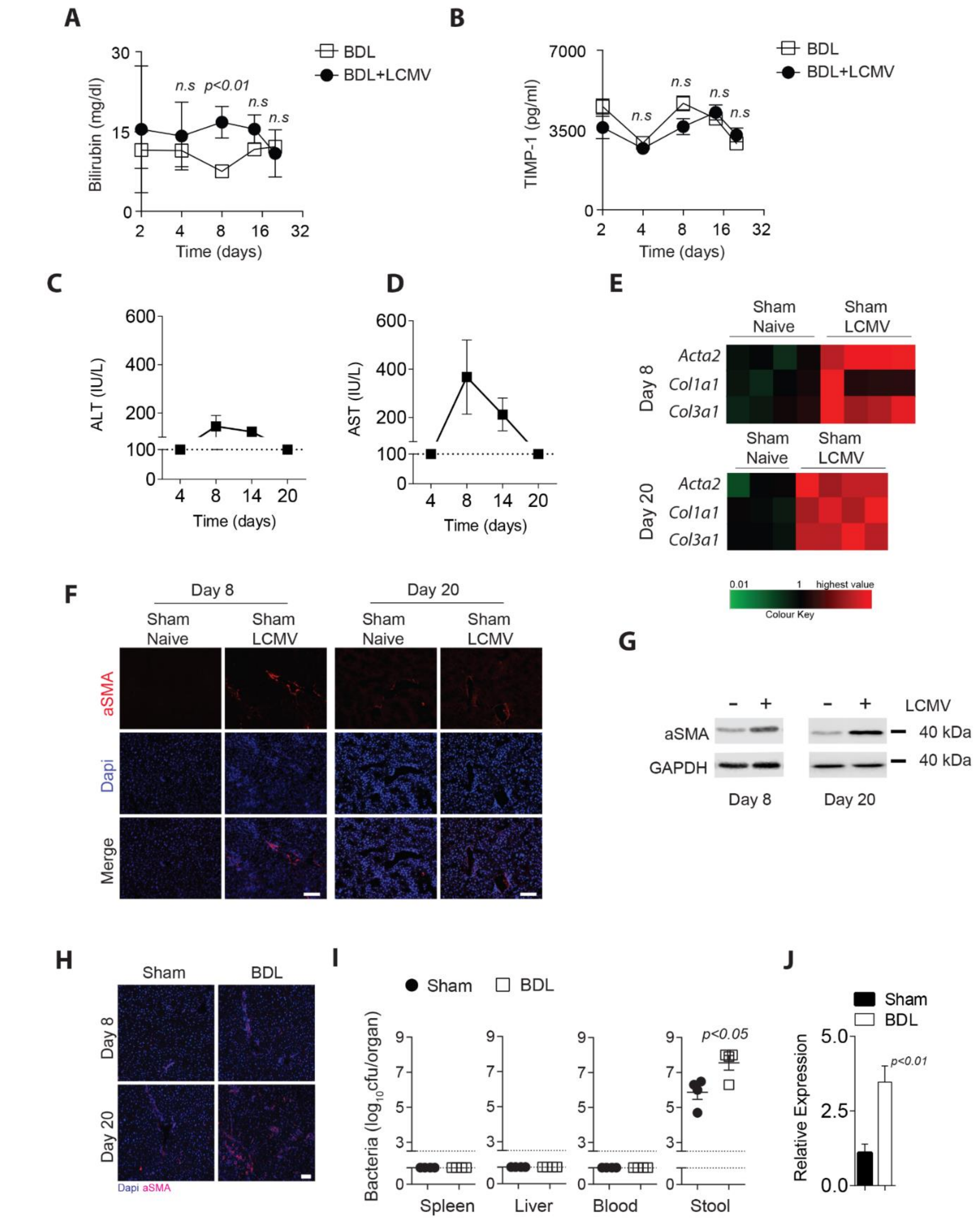

Supplementary Figure 1: Bile duct ligation results in liver damage. (A-B) Bile duct ligation was performed in C57Bl/6 mice. One day after the operation, mice were infected without or with 2x10^4 pfu of LCMV WE. **(A)** Bilirubin concentration was determined in the serum of infected and BDL treated animals (n=6). **(B)** TIMP-1 concentration was measured in the serum of LCMV infected BDL and control BDL mice (n=6). **(C and D)** One day after the sham operation, mice were infected with 2x10^4 pfu of LCMV WE. **(C)** ALT activity and **(D)** AST activity was measured over time in infected animals (n=3-4). **(E-G)** Sham mice were infected with 2x10^4 pfu of LCMV WE and gene expression was evaluated for Acta2, Col1a1, Col3a1 at day 8 and day 20 after infection (n=3-4). ..*Continued*

Continued…(F) Sections from snap frozen liver tissue harvested from mice following 8 days (upper panels) and 20 days (lower panels) p.i. were stained for α-SMA (red) and Dapi (blue). One representative of n=4 is shown, scale bar=100µm. **(G)** Protein samples were isolated from liver tissue of sham operated mice 8 days (left panels) and 20 days (right panels) after infection. α-SMA expression (upper panels) and GAPDH expression (lower panels) were determined (one representative immunoblot of n=4 is shown). Un-cropped western blot images are shown in supplementary Fig.7c. (H) Sham and BDL operated mice were infected with LCMV and liver tissue harvested after day 8 and day 20 and tissue sections were stained for α-SMA. One representative of n=4 is shown, scale bar=100µm. **(I)** Naïve Sham and BDL treated mice were sacrificed 24h after the procedure. Total bacterial load was determined in various organs (n=4). **(J)** *Ifnβ* gene expression was determined 2 days after BDL treatment in liver tissue of sham and BDL mice without infection (n=4).

Supplementary Figure 2

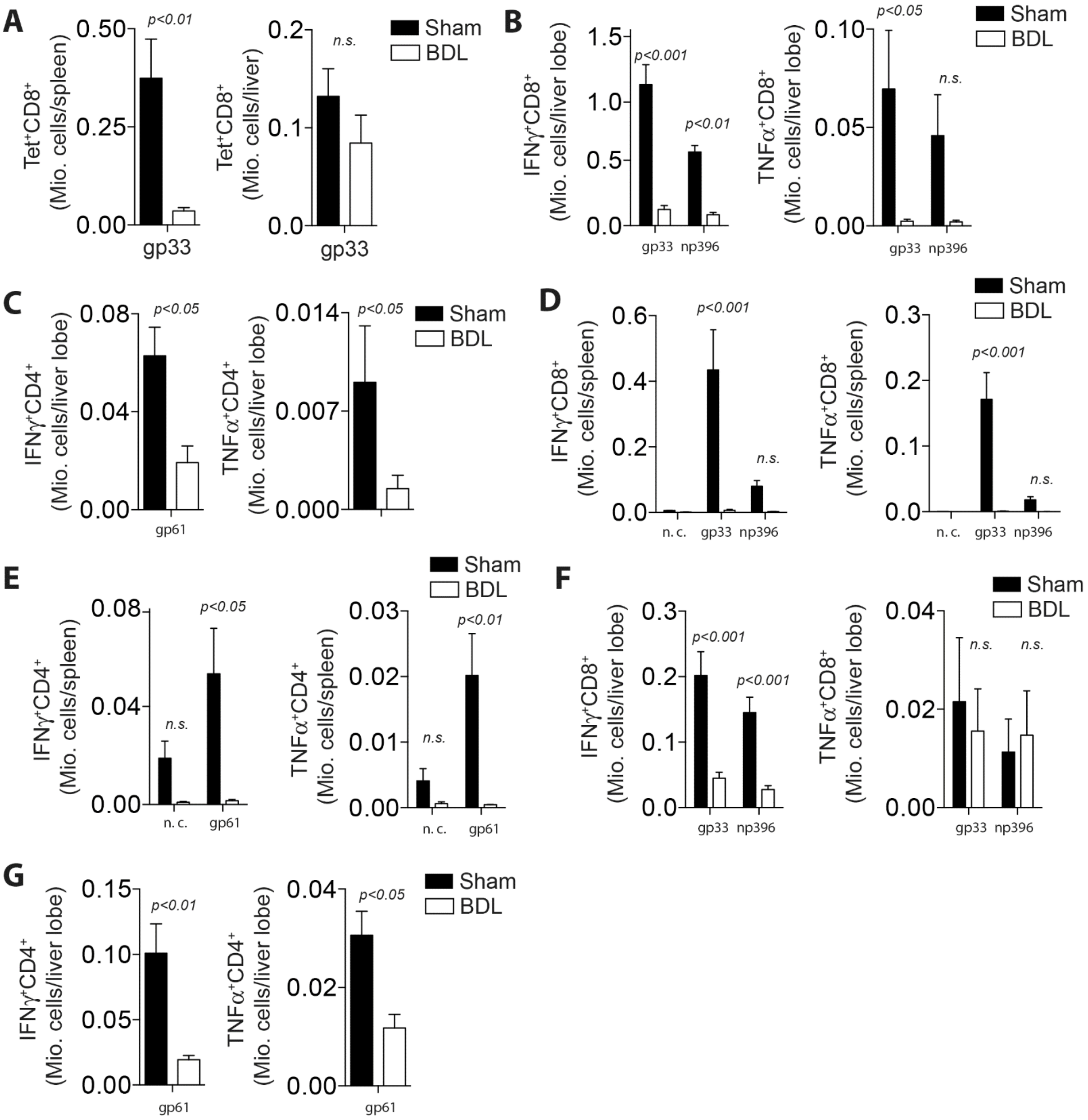

Supplementary Figure 2: Anti-viral T cell immunity is defective following BDL. (A-G) C57Bl/6 mice were either operated with BDL or Sham followed by infection with $2x10^4$ PFU of LCMV WE. **(A)** Virus specific T cells against gp33 were measured in spleen (left panel) and liver (right panel) tissue 20 days after infection (n=5-7). **(B)** 8 days after infection, single cell suspended liver cells were restimulated with the LCMV specific epitopes gp33 and np396. IFN-γ (left panel) and TNF- α (right panel) production was determined in $CD8^+$ T cells. **(C)** 8 days after infection single cell suspended liver cells were exposed to the MHC-II epitope gp61. IFN-γ (left panel) and TNF-α (right panel) production was determined in $CD4^+$ T cells. **(D)** 20 days after infection, single cell suspended splenocytes were restimulated with the LCMV specific epitopes gp33 and np396. IFN-γ (left panel) and TNF-α (right panel) production was determined in $CD8^+$ T cells. **(E)** 20 days after infection single cell suspended splenocytes were exposed to the MHC-II epitope gp61. IFN-γ (left panel) and TNF-α (right panel) production was determined in $CD4^+$ T cells. **(F)** 20 days after infection, single cell suspended liver cells were restimulated with the LCMV specific epitopes gp33 and np396. IFN-γ (left panel) and TNF-α (right panel) production was determined in $CD8^+$ T cells. **(G)** 20 days after infection single cell suspended liver cells were exposed to the MHC-II epitope gp61. IFN-γ (left panel) and TNF-α (right panel) production was determined in $CD4^+$ T cells.

Supplementary Figure 3

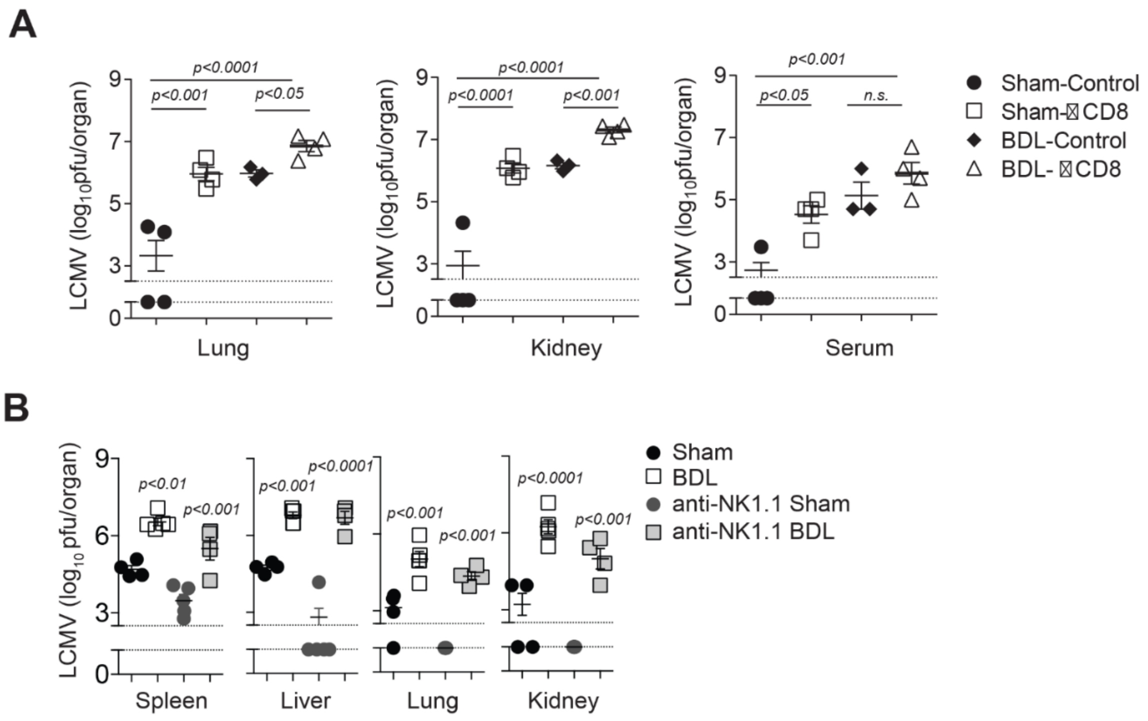

Supplementary Figure 3: NK cell depletion cannot rescue defective T cell immunity. (A) CD8 T cell were depleted in Sham and BDL mice and infected with LCMV WE. Virus titers were determined in the indicated organs 8 days after infection (n=3-4). **(B)** At day -1, mice were either operated with BDL or Sham followed by infection with $2x10^4$ pfu of LCMV WE at day 0. Anti-NK1.1 antibody was administered to C57Bl/6 mice at day -3 and day -1 of infection. Virus titers were measured in different organs as indicated 8 days after infection (n=4).

Supplementary Figure 4

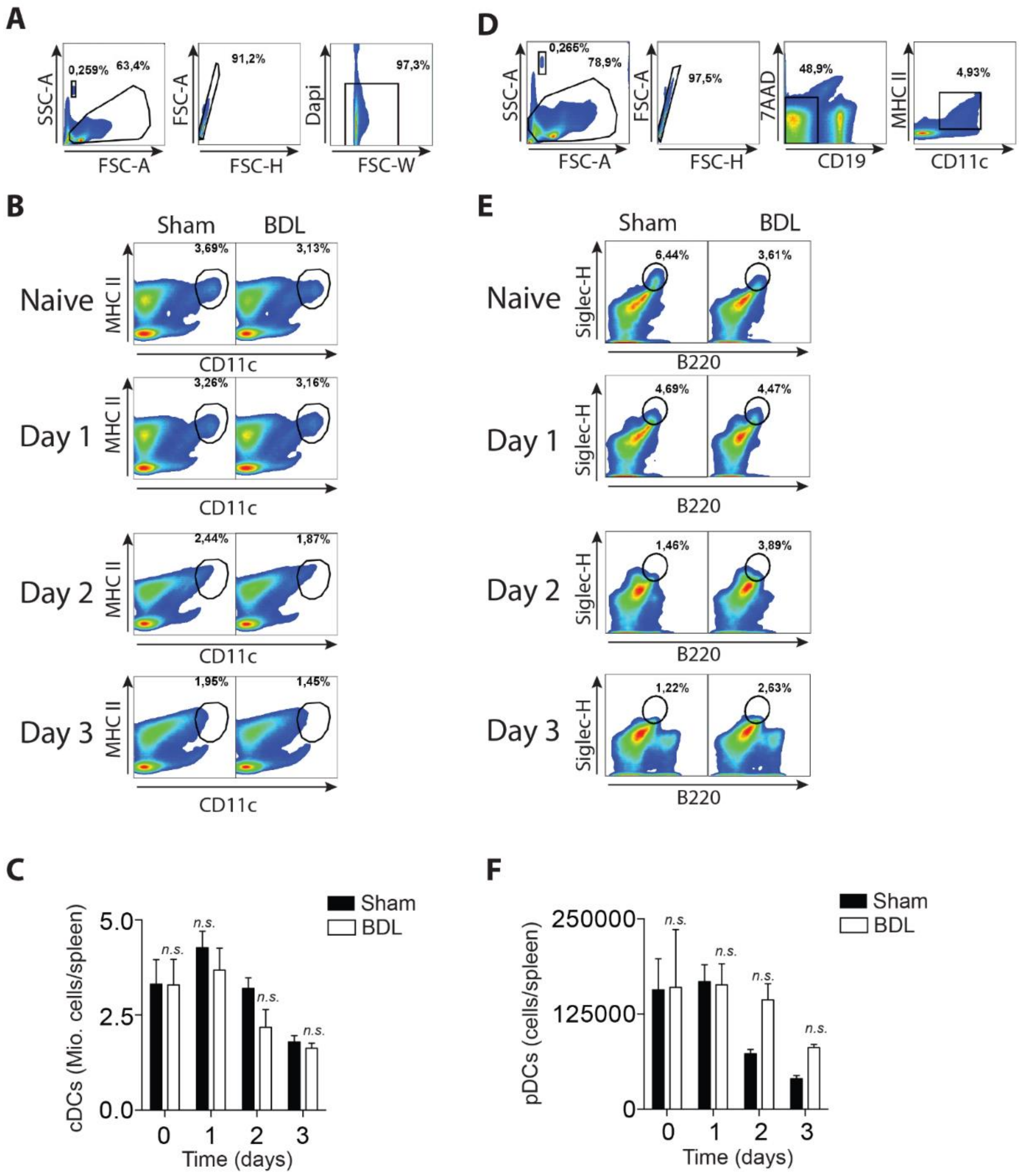

Supplementary Figure 4: Dendritic cell subsets are not reduced following BDL. (A-F) Sham and BDL treated mice were infected with $2x10^4$ pfu of LCMV WE. **(A)** Gating strategy to identify conventional dendritic cells is shown (One representative of n=7 is shown). **(B)** Contour plots indicating CD11c and MHC-II expression are illustrated in Sham or BDL treated animals at indicated time points (one representative of n=7 is shown). **(C)** Mean ± SEM of $CD11c^+MHC\text{-}II^+$ cell numbers are shown at indicated time points after Sham or BDL operation (n=7). **(D)** Gating strategy to identify plasmacytoid dendritic cells is shown (One representative of n=4 is shown). **(E)** Contour plots indicating B220 and Siglec-H expression are illustrated in Sham or BDL treated animals at indicated time points (one representative of n=4 is shown). **(F)** Mean ± SEM of Siglec-H^+B220^+ cell numbers are shown at indicated time points after Sham or BDL operation (n=4).

Supplementary Figure 5

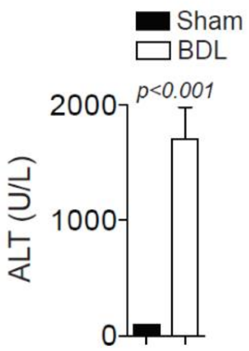

Supplementary Figure 5: Liver enzymes are increased following BDL.

ALT activity was measured in serum from C57Bl/6 mice 24h after either BDL or Sham operation (n=6).

Supplementary Figure 6

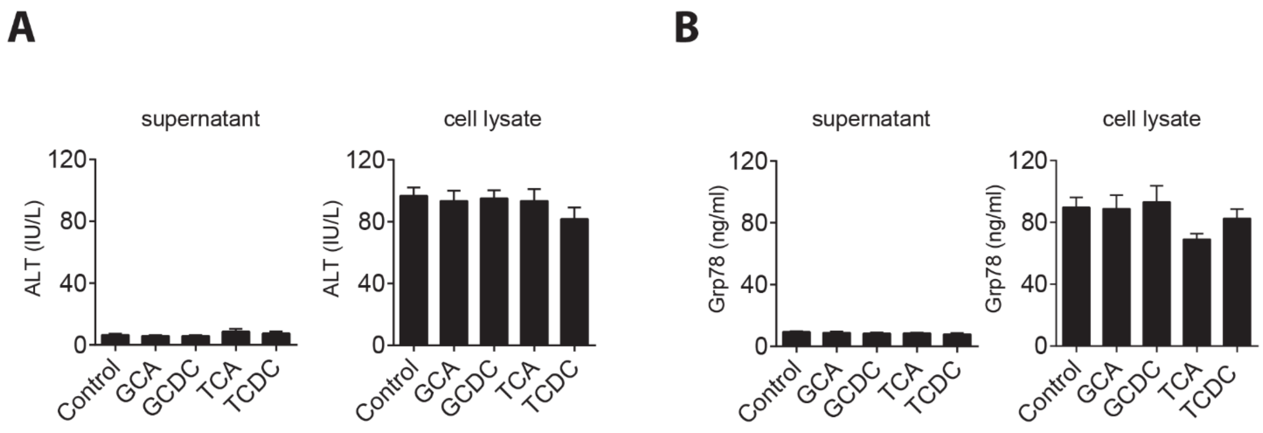

Supplementary Figure 6: GRP78 is a biomarker of liver damage. (A) ALT and **(B)** GRP78 levels in the supernatant and cell lysate was measured from HepG2 cells treated with bile acids GCA (100 μM), GCDC (100 μM), TCA (500 μM) and TCDC (100 μM) for 24 h (n=6).

Supplementary Figure 7

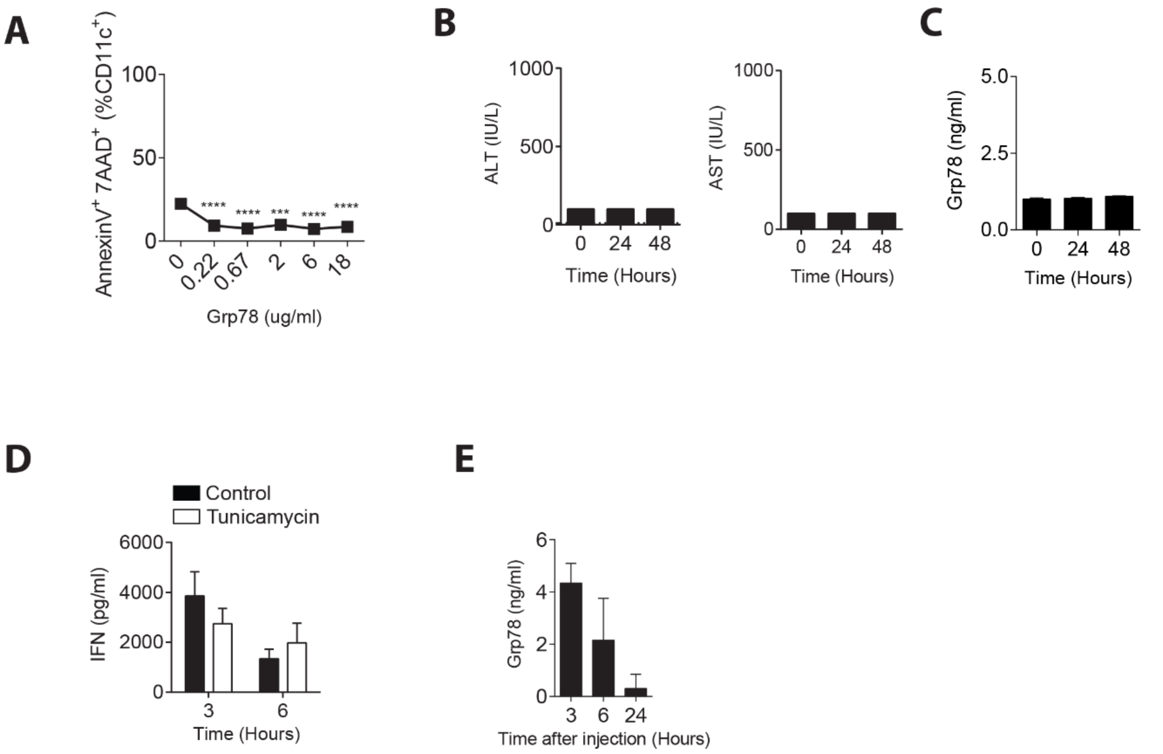

Supplementary Figure 7: (A) To assess cell death, BMDCs were stained with Annexin V and 7AAD 24h after GRP78 stimulation at indicated concentrations (n=4). **(B)** Mice were administered with 20nm Tunicamycin day -1. ALT and AST was determined 24 and 48 h after Tunicamycin injection (n=3). **(C)** GRP78 levels in the serum samples were assessed from the samples as in Fig S5H (n=3)**. (D)** Mice were injected with 25 ug pI:C 48h after Tunicamycin treatment, IFN alpha was measured in the serum samples at indicated time points (n=3). **(E)** GRP78 (25 ug) was administered via intraperitoneal injection day -1 and day 0. Serum samples were assessed for GRP78 concentration at indicated time points after the second dose (n=4).

Supplementary Figure. 8.

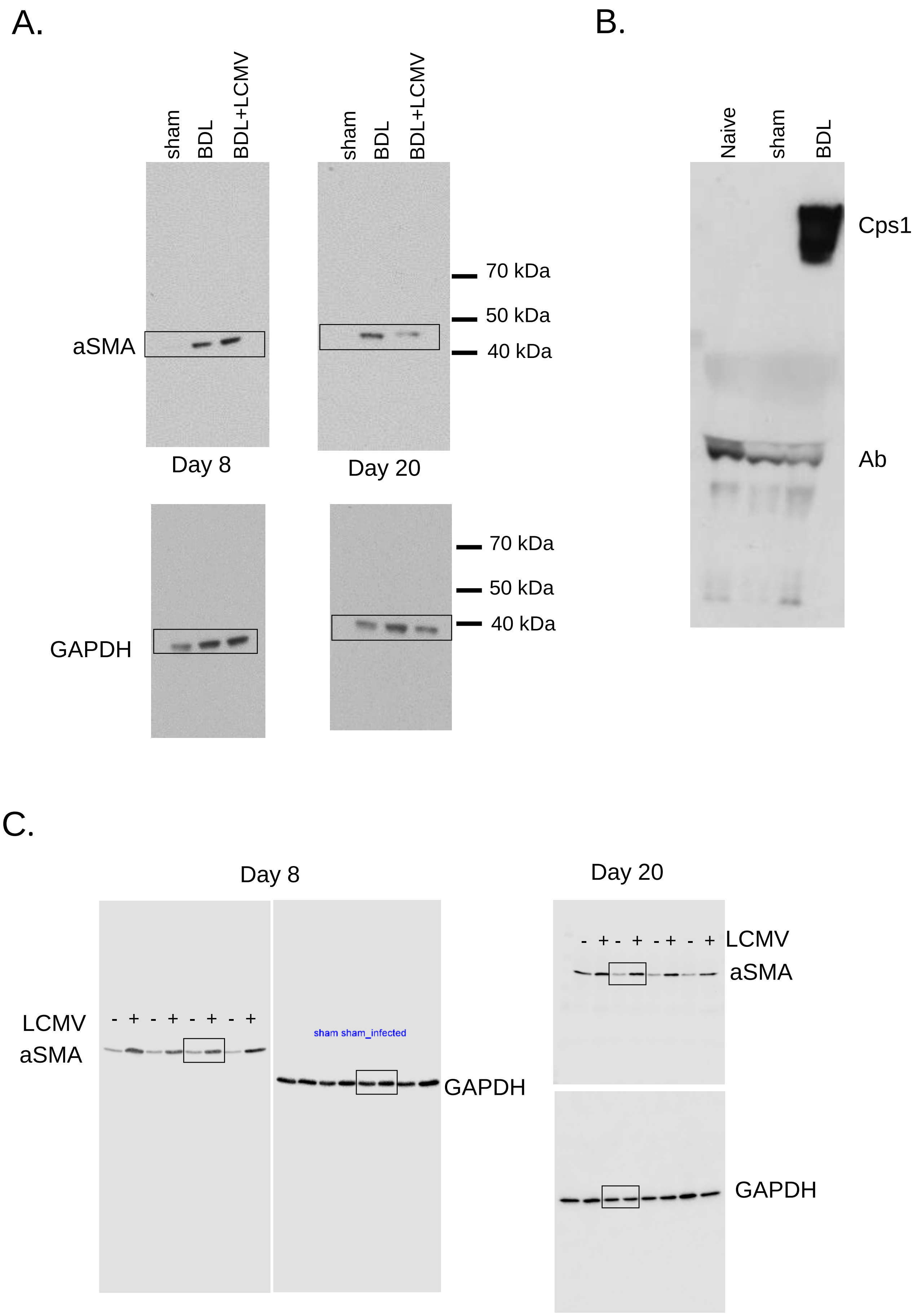

Supplementary Figure 8: Uncropped western blot images . **(A)** Uncropped Western blot (WB) image for Figure 1E. Protein samples were isolated from liver tissue of naïve mice and animals following BDL treatment 8 days (left panels) and 20 days (right panels) after or without infection (as indicated). α-SMA expression (upper panels) and GAPDH expression (lower panels) were determined (one representative of n=6 is shown). **(B)** Uncropped WB image for Figure 4E. WB analyses of whole serum samples harvested from naïve, Sham or BDL animals following immunostaining with anti-CPS-1 (carbamoyl phosphate synthetase) and an anti-mouse secondary antibody is shown (one representative of n=4 is shown). (C) Uncropped Western blot (WB) image for Supplementary Figure 1G.

Supplementary Table S1:

	Sham	BDL	P value
T-w-MCA	20,45±15,03	188,0 ± 6,859	< 0,0001
T-beta-MCA	10,95±6,837	115,4 ± 8,949	< 0,0001
THCA	0,1167±0,1167	0,6100 ± 0,1773	0.0669
TCA	38,77±26,00	605,8 ± 53,84	< 0,0001
TUDCA	0,1833±0,1138	0,1900 ± 0,02769	0.9441
THDCA	0,1848±0,1421	0,2368 ± 0,04801	0.6826
TCDCA	0,5167±0,4199	0,3200 ± 0,02906	0.5483
TDCA	0,4167±0,2330	0,1700 ± 0,03958	0.2004
TLCA	0,01667±0,01667	0,0 ± 0,0	0.2071
T-alpha-MCA	2,317±1,173	0,0 ± 0,0	0.0205
GLCA	0,0±0,0	0,1500 ± 0,1003	0.2724
GDCA	7,417±2,915	4,100 ± 1,440	0.2719
GCDCA	33,78±17,22	49,34 ± 4,841	0.3007
GHDCA	0,0±0,0	0,1500 ± 0,1003	0.2724
GUDCA	0,4667±0,2963	0,2400 ± 0,1661	0.4798
GCA	0,6833±0,3902	53,86 ± 11,02	0.0025
G-beta-MCA	0,006667±0,006667	0,0110 ± 0,003145	0.5163
alpha-MCA	0,5667±0,4072	0,0100 ± 0,0100	0.0923
beta-MCA	5,550±3,999	9,290 ± 0,9373	0.2709
HCA	0,06667±0,04944	0,0400 ± 0,01633	0.5457
CA	6,400±2,974	1,500 ± 0,6482	0.0608
MDCA	0,0500±0,0500	0,0 ± 0,0	0.2071
UDCA	0,2833±0,2257	0,0 ± 0,0	0.119
HDCA	0,1167±0,08333	0,0100 ± 0,0100	0.1196
CDCA	0,2500±0,1310	0,0 ± 0,0	0.0243
DCA	1,150±0,6076	0,0500 ± 0,01667	0.0313
Taurin-conjugated	73,93±49,74	910,7 ± 64,11	< 0,0001
Glycin-conjugated	42,33±20,21	107,9 ± 15,67	0.0226
Unconjugated	16,08±8,430	15,08 ± 1,695	0.8839

Supplementary Table S1: Bile acid composition in BDL treated and Sham control mice. Bile acid profiles were determined in sera from mice 24h after BDL or Sham control operation (n=3).

Supplementary Table S2

Protein names	Gene names	mean BDL	mean sham	ratio BDL/sham	Welch's t-test q-value BDL_Sham	Welch's t-test Difference BDL_Sham
L-lactate dehydrogenase A chain	Ldha	163775408.9	NA	NA	0	6.26028
Carbamoyl-phosphate synthase [ammonia], mitochondrial	Cps1	416445916.1	NA	NA	0	6.15769
Betaine--homocysteine S-methyltransferase 1	Bhmt	559528112.3	NA	NA	0	6.13603
3-ketoacyl-CoA thiolase, mitochondrial	Acaa2	138745538.6	NA	NA	0	5.87326
Fructose-bisphosphate aldolase B	Aldob	560860161.2	19686411.24	28.49	0	5.49331
Ornithine carbamoyltransferase, mitochondrial	Otc	116558320.5	NA	NA	0	5.05303
Sorbitol dehydrogenase	Sord	72666400.98	NA	NA	0	4.90354
Dihydropyrimidinase	Dpys	178958608	NA	NA	0	4.63343
Argininosuccinate lyase	Asl	98358809.52	NA	NA	0	4.57606
Arginase-1	Arg1	54409356.44	NA	NA	0	4.51359
Retinal dehydrogenase 1	Aldh1a1	78583993.29	NA	NA	0	4.44253
Fructose-1,6-bisphosphatase 1	Fbp1	100489679.7	NA	NA	0	4.19457
Polymeric immunoglobulin receptor;Secretory component	Pigr	134578107.1	27370149.9	4.92	0	4.15465
Adenosylhomocysteinase	Ahcy	62416083.01	NA	NA	0	4.13163
Isocitrate dehydrogenase [NADP] cytoplasmic	Idh1	55427207.38	NA	NA	0	4.04516
Homogentisate 1,2-dioxygenase	Hgd	59708471.87	NA	NA	0	4.00444
ATP synthase subunit beta, mitochondrial	Atp5b	40340838.24	NA	NA	0	3.60073
Selenium-binding protein 2;Selenium-binding protein 1	Selenbp2;Selenbp1	57833508.41	NA	NA	0	3.48698
Fumarylacetoacetase	Fah	81384943.42	NA	NA	0	3.36571
78 kDa glucose-regulated protein	Hspa5	59393507.42	5145003.859	11.54	0	3.15033
Glycogen phosphorylase, liver form	Pygl	40879371.71	NA	NA	9.30E-05	3.13201
Glutamate dehydrogenase 1, mitochondrial	Glud1	54891741.82	NA	NA	0	3.12039
ATP synthase subunit alpha, mitochondrial	Atp5a1	64808675.94	NA	NA	0.000769231	3.08111
Alcohol dehydrogenase 1	Adh1	64131331.97	NA	NA	0	3.06334
Proteasome subunit alpha type-1	Psma1	27194176.47	7700389.547	3.53	0	3.03339
Malate dehydrogenase, cytoplasmic	Mdh1	41506359.71	NA	NA	0	3.01499
Catalase	Cat	78769017.71	NA	NA	0	2.94731
Carbonic anhydrase 2	Ca2	182303844.8	27050196.24	6.74	0	2.93389
Aspartate aminotransferase, mitochondrial	Got2	41545017.69	NA	NA	0	2.91215
Aldehyde dehydrogenase family 8 member A1	Aldh8a1	23243017.54	NA	NA	0.00032	2.91058
Cytosol aminopeptidase	Lap3	41478094.09	7165818.143	5.79	0	2.84397
Heat shock protein HSP 90-alpha	Hsp90aa1	18031508.79	NA	NA	9.52E-05	2.82773
4-hydroxyphenylpyruvate dioxygenase	Hpd	19481436.64	NA	NA	0	2.82707
Cytosolic 10-formyltetrahydrofolate dehydrogenase	Aldh1l1	43928232.3	NA	NA	0	2.68512
Heat shock cognate 71 kDa protein;Heat shock-related 70 kDa protein 2	Hspa8;Hspa2	46668693.09	6547622.261	7.13	0.00026087	2.59494
Cystathionine gamma-lyase	Cth	33496221.34	NA	NA	0.000105263	2.57513
Argininosuccinate synthase	Ass1	30932190.93	NA	NA	0.000102564	2.51592
Ig alpha chain C region		216512611.4	36357547.38	5.96	0	2.42428
Glycine N-methyltransferase	Gnmt	47307412.98	NA	NA	0	2.40794

SupplementaryTable S2: Proteins identified by mass spectrometry in serum fractions. Differentially abundant proteins in sera from 6 BDL and 6 sham mice were detected by quantitative mass spectrometry. Protein identification data as well as mean normalized intensities from label-free quantitative mass spectrometric measurements are given. Furthermore, q-values calculated from Welch's t-tests are reported.

Supplementary Table 2 continued

Alpha-enolase	Eno1	17668615.44	NA	NA	0.00407273	2.24355
Urocanate hydratase	Uroc1	22619330.21	NA	NA	0.0001	2.11198
Transketolase	Tkt	24942954.85	4970414.869	5.02	9.09E-05	2.08872
Sarcosine dehydrogenase, mitochondrial	Sardh	22388345.14	NA	NA	0.00544828	2.02063
Aspartate aminotransferase, cytoplasmic	Got1	23150063.45	NA	NA	0.0168824	1.96531
UTP--glucose-1-phosphate uridylyltransferase	Ugp2	10717739.37	NA	NA	0	1.86084
Apolipoprotein E	Apoe	5619735434	1561643635	3.60	9.76E-05	1.84392
Glyceraldehyde-3-phosphate dehydrogenase	Gapdh	16514776.9	6725467.316	2.46	0.00025	1.8406
60 kDa heat shock protein, mitochondrial	Hspd1	14881050.62	NA	NA	0	1.81614
Actin, cytoplasmic 2;Actin, cytoplasmic 2, N-terminally processed;Actin, cytoplasmic 1;Actin, cytoplasmic 1, N-terminally processed	Actg1;Actb	747731129.9	211050551.7	3.54	0	1.7955
Cytochrome P450 2F2	Cyp2f2	23991121.96	NA	NA	0.000255319	1.72932
Peroxisomal acyl-coenzyme A oxidase 1	Acox1	15875938.2	NA	NA	0.000244898	1.58157
Lipopolysaccharide-binding protein	Lbp	33175448.43	10355983.2	3.20	0.000784314	1.53041
Nucleoside diphosphate kinase A	Nme1	29206585.21	NA	NA	0.035662	1.48382
Isovaleryl-CoA dehydrogenase, mitochondrial	Ivd	18113202.28	NA	NA	0.0363288	1.40095
Alpha-aminoadipic semialdehyde dehydrogenase	Aldh7a1	15748300.73	NA	NA	0.00773333	1.39455
Peroxiredoxin-2	Prdx2	13955790.62	4548855.097	3.07	0.00745763	1.33081
Dimethylglycine dehydrogenase, mitochondrial	Dmgdh	12586397.23	NA	NA	0.00333333	1.3217
Aldehyde dehydrogenase, mitochondrial	Aldh2	13508258.96	NA	NA	0.00339623	1.31148
Pyruvate carboxylase, mitochondrial	Pc	7037875.049	NA	NA	8.89E-05	1.26246
Immunoglobulin J chain	Igj	26672733.85	9700537.022	2.75	0.0105	1.22498
Fructose-bisphosphate aldolase A	Aldoa	52678048.26	24565691.88	2.14	0.0115152	1.07588
Vinculin	Vcl	8815737.18	NA	NA	0.00780328	0.986004
Aldehyde oxidase 3	Aox3	36630567.67	NA	NA	0.00435714	0.892914
Ceruloplasmin	Cp	2265661325	1247078071	1.82	0.0106032	0.793361
Phosphatidylcholine-sterol acyltransferase	Lcat	51723222.63	30324664.88	1.71	0.0495263	0.764956
Apolipoprotein A-IV	Apoa4	2078788893	1312516255	1.58	0.0162985	0.712022
Serum amyloid A-4 protein	Saa4	79584230.23	117012022.2	0.68	0.0361667	-0.544148
Pigment epithelium-derived factor	Serpinf1	22490119.72	33469235.9	0.67	0.0116308	-0.58909
Complement factor D	Cfd	74591683.24	118639772.9	0.63	0.0467733	-0.627148
Gelsolin	Gsn	779440852.7	1243586939	0.63	0.0181714	-0.676261
Mannose-binding protein C	Mbl2	27740677.74	47450676.9	0.58	0.0406486	-0.727659
Hemopexin	Hpx	6490226540	10657182311	0.61	0.018087	-0.771365
Angiotensinogen;Angiotensin-1;Angiotensin-2;Angiotensin-3;Angiotensin-4;Angiotensin 1-9;Angiotensin 1-7;Angiotensin 1-5;Angiotensin 1-4	Agt	34092748.67	59415099.98	0.57	0.00825806	-0.881438
Insulin-like growth factor-binding protein complex acid labile subunit	Igfals	37536003.03	73404547.51	0.51	0.0045614	-1.19237
Vitronectin	Vtn	94348959	298191174.5	0.32	0	-1.67477
Haptoglobin;Haptoglobin alpha chain;Haptoglobin beta chain	Hp	396783342.5	4654175263	0.09	0	-3.66662

Supplementary Table S3

Characteristics	Cohort A	Cohort B
	ALT<br><50 U/L (n=57)	ALT<br>>50 U/L (n=20)
Healthy Control	3	0
HBV	22	2
HBV, Liver Cirrhosis	3	1
HBV, NASH	4	6
NASH, Liver Cirrhosis	0	1
HBV, NASH, Liver Cirrhosis	2	1
HCV	13	0
HCV, Liver cirrhosis	3	1
HCV, NASH	0	1
Others	7	7

Supplementary Table S3: Patient Cohort with liver damage. Patient Cohort of 77 subjects were selected according to increased liver pathology markers.
